# The clinical and molecular landscape of thalamic glioma

**DOI:** 10.1093/neuonc/noag146

**Published:** 2026-06-30

**Authors:** Linpeng Zhang, Hesong Wang, Guangrui Huang, Xueling Qi, Angel M Carcaboso, Chen Wang, Jing Feng, Song Han, Zhilong Cheng, Zhong Ma, Yakun Yang, Heng Xu, Minshuo Chen, Yongli Li, Xufei Zhang, Gang Cui, Jiacheng Lyu, Zejun Duan, Anlong Xu, Changxiang Yan

**Affiliations:** Department of Neurosurgery, Sanbo Brain Hospital, Capital Medical University, Beijing, China; School of Life Sciences, Beijing University of Chinese Medicine, Beijing, China; School of Life Sciences, Beijing University of Chinese Medicine, Beijing, China; Institute for Translational Oncology, Beijing University of Chinese Medicine, Beijing, China; Department of Pathology, Sanbo Brain Hospital, Capital Medical University, Beijing, China; SJD Pediatric Cancer Center Barcelona, Hospital Sant Joan de Deu, Barcelona, Spain; Institut de Recerca Sant Joan de Deu, Barcelona, Spain; Department of Neurosurgery, Sanbo Brain Hospital, Capital Medical University, Beijing, China; Department of Pathology, Sanbo Brain Hospital, Capital Medical University, Beijing, China; Department of Neurosurgery, Sanbo Brain Hospital, Capital Medical University, Beijing, China; Department of Neurosurgery, Sanbo Brain Hospital, Capital Medical University, Beijing, China; Department of Pathology, Sanbo Brain Hospital, Capital Medical University, Beijing, China; Department of Neurosurgery, Sanbo Brain Hospital, Capital Medical University, Beijing, China; School of Life Sciences, Beijing University of Chinese Medicine, Beijing, China; School of Life Sciences, Beijing University of Chinese Medicine, Beijing, China; Department of Pathology, Sanbo Brain Hospital, Capital Medical University, Beijing, China; Department of Radiology, Sanbo Brain Hospital, Capital Medical University, Beijing, China; Department of Neurosurgery, Sanbo Brain Hospital, Capital Medical University, Beijing, China; Department of Nursing Unit, Sanbo Brain Hospital, Capital Medical University, Beijing, China; Department of Pathology, Sanbo Brain Hospital, Capital Medical University, Beijing, China; School of Life Sciences, Beijing University of Chinese Medicine, Beijing, China; Institute for Translational Oncology, Beijing University of Chinese Medicine, Beijing, China; Hong Kong Institute of Advanced Studies, Sun Yat-sen University, Hong Kong, China; Department of Neurosurgery, Sanbo Brain Hospital, Capital Medical University, Beijing, China

**Keywords:** hemiparesis, hydrocephalus, mutation landscape, surgical treatment, thalamic glioma

## Abstract

**Background:**

Thalamic gliomas (TGs) remain a formidable clinical challenge for accurate diagnosis and effective therapy. This study aims to refine molecular diagnosis and surgical management of TGs by integrating clinicopathological and multiomics data.

**Methods:**

We analyzed 106 TGs, comprising 65 diffuse midline glioma (DMG) and 41 non-DMG, using genomic profiling, single-cell RNA sequencing, and orthogonal assays. Surgical outcomes were evaluated using rigorous causal inference frameworks, including propensity score matching and difference-in-differences analyses.

**Results:**

Genomic profiling revealed striking mutual exclusivity between *H3F3A* mutations and *CDKN2A/B* (9p21) loss. Single-cell and orthogonal assays validated that 9p21 loss emerged as a highly specific adjunctive marker favoring non-DMG over DMG. Causal inference analyses consistently demonstrated that microsurgical third ventriculostomy (mTV) confers profound protection against postoperative hydrocephalus (risk reduction >60%). Multivariate analysis identified the transfrontal approach as an independent predictor of postoperative motor deficits. Survival analysis showed that the median overall survival of this cohort following surgical resection was 21.0 months. Extent of resection and tumor mutational burden emerged as the principal independent determinants of prognosis, whereas postoperative Karnofsky performance status showed survival stratification in univariable/subgroup analyses but was not independently significant in the multivariable model.

**Conclusions:**

In this 106-patient cohort study, we delineated the somatic mutational landscape of TG and showed that 9p21 loss is mutually exclusive with *H3F3A* mutations, providing a highly specific adjunctive marker for TG. We further demonstrated that maximal surgical resection confers a survival benefit and intraoperative mTV serves as a standardized treatment workflow to optimize care for TG.

Key Points

- *H3F3A* mutation and *CDKN2A/B* co-deletion are largely mutually exclusive in TGs.
- Microsurgical third ventriculostomy reduces hydrocephalus risk, while the transfrontal approach increases hemiparesis risk in TGs.
- Extensive resection improves overall survival in TGs.

Importance of the Study

TGs are rare, surgically challenging tumors adjacent to eloquent motor pathways and cerebrospinal fluid outflow tracts, frequently presenting with hydrocephalus and motor deficits. In a resection-based cohort of 106 patients, we integrated clinicoradiological data with targeted panel sequencing and single-cell transcriptomic reference analyses. We describe a mutational landscape in which *H3F3A* alteration and *CDKN2A/B* co-deletion are largely mutually exclusive, supporting biological divergence between diffuse midline glioma (DMG) and non-DMG tumors. We identify predictors of postoperative hydrocephalus and show that microsurgical third ventriculostomy is associated with reduced cerebrospinal fluid complications. Preoperative hemiparesis was the strongest correlate of motor worsening, and greater cytoreduction correlated with improved survival.

Thalamic gliomas (TGs) are deep-seated tumors abutting the internal capsule, midbrain, and other critical projection pathways and remain among the most challenging entities in neurosurgical oncology. TGs account for approximately 1-5% of intracranial gliomas. They commonly present with raised intracranial pressure, pyramidal tract signs, and cognitive or consciousness disturbance, and their overall prognosis remains poor.^1^ According to the 5th Edition of the World Health Organization Classification of Tumors of the Central Nervous System (WHO CNS5), H3K27-altered diffuse midline glioma (DMG) is defined as a distinct molecular entity and assigned WHO grade 4 irrespective of histology. The thalamus represents a key anatomic site for this subgroup.^2^

Optimal management remains debated. Available data suggest that, in carefully selected unilateral TGs, greater extent of resection (EOR) may be associated with improved survival; however, evidence specifically supporting this in thalamic H3K27-altered DMG remains limited and evolving. Stereotactic biopsy is generally reserved for diffuse bilateral thalamic disease.^3^ In practice, complete or near-complete resection is often technically prohibitive, and biopsy-centered strategies remain common for establishing a molecular diagnosis. Systematic analyses examining how surgical corridor, EOR, and local anatomy influence postoperative complications, particularly hydrocephalus and hemiparesis, remain scarce.^3-5^ The literature is further limited by small, heterogeneous cohorts and by the restricted tissue yield from biopsy specimens, which impedes comprehensive multiomics characterization and functional pathway studies.^6-8^

To address these gaps, we retrospectively analyzed 106 patients with TGs treated with microsurgical resection. We integrated clinical presentation, imaging phenotype, and pathological and molecular features; evaluated treatment pathways and survival; and quantified risk factors for postoperative hydrocephalus and hemiparesis. Targeted exonic sequencing was performed to define the mutational landscape. This resection-based cohort provides clinically actionable evidence to inform surgical decision-making, perioperative risk management, and molecularly stratified care.

## Methods

### Cohort and Ethics

We retrospectively identified 106 consecutive patients with newly diagnosed unilateral TG who underwent microsurgical resection at Sanbo Brain Hospital, Capital Medical University, between June 1, 2015 and December 31, 2024. Eligibility required a dominant lesion centered in 1 thalamus. The study was approved by the institutional ethics committee, conducted in accordance with the Declaration of Helsinki, and all patients gave written informed consent.

### Clinical Variables, Motor Assessment, and Follow-Up

From medical records and follow-up, we collected age, sex, presenting symptoms, neurological examination, Karnofsky performance status (KPS), surgical corridor, postoperative KPS, and early postoperative neurological status. Limb motor strength was graded bilaterally using the Medical Research Council (MRC) 0-5 scale pre- and postoperatively; a patient-level “worst” MRC grade was defined as the minimum of bilateral score and ΔMRC as the postoperative minus the preoperative worst grade. Pre- and postoperative hemiparesis were coded from neurological examination (detailed motor assessment protocol in Supplementary Methods S1.1). Pathology/molecular findings, adjuvant therapy, and survival status were updated to July 2025. Early postoperative CT was obtained within 24 h, postoperative MRI within 1 month when feasible, and follow-up imaging every 4-6 months thereafter.

### Neuroimaging and Radiological Assessment

MRI protocols included 3D fluid-attenuated inversion recovery (FLAIR), contrast-enhanced 3D T1-weighted imaging, diffusion-weighted imaging/apparent diffusion coefficient and conventional T1/T2; selected patients additionally underwent DTI and cardiac-gated phase-contrast cine MRI of the cerebral aqueduct for cerebrospinal fluid (CSF) flow assessment (Supplementary Methods S1.2). Two neuroradiologists independently assessed tumor volume and maximum diameter, laterality and topography, extension (including midline/contralateral spread), cystic change, peritumoral edema, midline shift, tract involvement, CSF flow abnormality, hydrocephalus and distant dissemination. Peritumoral edema was summarized by an edema index (edema ­volume + tumor volume)/tumor volume, categorized as none (1.0), mild (1.0-1.5), or severe (>1.5). Hydrocephalus was defined as ventriculomegaly without external CSF drainage and an Evans ratio of >0.33; postoperative hydrocephalus within 1 month was assessed on serial CT/MR.

### Surgery and EOR

Surgical corridors were chosen according to lesion laterality, growth direction, and relationship to major white-matter tracts. Three transcortical-transventricular routes were used (1) frontal trans-lateral ventricle, (2) frontal trans-lateral ventricle with microsurgical third ventriculostomy (mTV), and (3) lateral ventricle trigone approach. EOR was determined by 2 neuroradiologists from early postoperative CT and contrast-enhanced/FLAIR MRI within 1 month and classified as gross total resection (GTR), subtotal resection (STR, 80-90%), or partial resection (PR, <80%).

### Pathology

All cases were diagnosed by a neuropathologist and reviewed by a senior neuropathologist according to the 2021 WHO CNS5 classification. Immunohistochemistry (IHC) and molecular tests were performed as clinically indicated.

### Targeted Sequencing and Variant Analysis

Tumor DNA was analyzed using a hybrid-capture targeted sequencing panel covering recurrently altered glioma genes. Reads were quality-controlled (FastQC/fastp), aligned to GRCh37 with BWA-MEM2, duplicate-marked with Picard, and somatic single-nucleotide variants (SNVs)/indels were called by GATK Mutect2 in tumor-only mode with a panel of normals and gnomAD germline reference, followed by orientation-bias and alignment-artifact filtering and Variant Effect Predictor annotation. High-confidence variants required depth ≥ 100 and variant allele frequency (VAF) ≥ 0.03; downstream mutation-level analyses applied depth ≥ 30, ≥ 5 alternate reads, and VAF ≥ 0.05. Known hypermutable genes (eg, MUC16, TTN) were excluded; TERT promoter variants were retained. TMB was calculated as protein-altering coding variants per megabase of captured sequence. Copy-number alterations (CNVs) were inferred from panel read-depth using the GATK CNV workflow (CollectReadCounts—DenoiseReadCounts—ModelSegments—CallCopyRatioSegments); per-gene absolute copy numbers were derived from length-weighted segment log2 ratios against a diploid baseline, with AMP defined as CN > 3 and DEL as CN < 1. Full pipeline parameters, thresholds, and panel-summary harmonization for cases are provided in Supplementary Methods S1.3-S1.8.

### Molecular Classification and Co-Alteration Analysis

Integrated SNV/indel and CNV calls were visualized as oncoplots stratified by age (pediatric vs adult) and diagnosis (DMG vs non-DMG) using maftools. Pairwise co-alteration between recurrent drivers was tested by Fisher’s exact tests with Benjamini-Hochberg adjustment and summarized as log2(observed/expected). Tumors were classified into 3 mutually exclusive molecular axes: H3K27 (H3F3A K27M), 9p21/cell-cycle (CDKN2A/B deletion without H3F3A mutation), and neither; RTK/PI3K pathway was coded as altered if any alteration was present in EGFR, NF1, PIK3CA, PIK3R1, PTEN, AKT1, RICTOR, or FGFR1. The diagnostic performance of 9p21 loss for distinguishing non-DMG from DMG was summarized by sensitivity, specificity, positive predictive value (PPV)/negative predictive value (NPV), and likelihood ratios with exact 95% CIs (Supplementary Methods S1.9-S1.12).

### Single-Cell RNA Sequencing

Fresh tumor samples from 1 unilateral thalamic DMG and 1 unilateral thalamic non-DMG underwent droplet-based scRNA-seq on the 10× Genomics platform. Data were processed with CellBender, Seurat v5 (SCTransform, RPCA integration, and graph-based clustering), and DoubletFinder, and cell types were annotated using canonical markers. Malignant cells were distinguished from microenvironmental cells by large-scale CNAs inferred with inferCNV and copyKAT. To quantify chr9:21-23 Mb (9p21) loss at single-cell resolution, we derived window-level Δlog2 metrics (fraction of malignant cells below predefined thresholds in raw and core windows) and run-length/core-4 anchored metrics across 5 core genes (KLHL9, MTAP, CDKN2A, CDKN2B-AS1, and CDKN2B). Quality-control thresholds, integration settings, marker panels, and metric definitions are provided in Supplementary Methods S1.13-S1.18.

### Histological Validation

H3K27 status was validated by IHC, and 9p21-region copy-number status was assessed using the 9p21/CEP9 interphase fluorescence in situ hybridization (FISH) ratio.

### Statistical Analysis

Continuous variables were compared with the Mann-Whitney *U* or *t* test and categorical variables with Fisher’s exact or χ² tests. Overall survival (OS) was estimated by Kaplan-Meier method and compared using log-rank tests. Independent prognostic factors were identified with multivariable Cox proportional hazards models.

For postoperative hydrocephalus, candidate predictors were first screened by stability selection with least absolute shrinkage and selection operator (LASSO) logistic regression (1000 iterations, 80% subsampling, cross-validated penalty) and then entered into a multivariable Firth penalized logistic regression to accommodate the rare-event setting; discrimination was summarized by area under the receiver operating characteristic curve (AUC) (DeLong 95% CIs, bootstrap optimism correction) and calibration by logistic recalibration (calibration intercept and slope), calibration plots, and Brier score. To estimate the causal effect of mTV, we combined (1) propensity-score analysis with scores estimated by ridge-penalized logistic regression (main specification), standard logistic regression, and covariate-balancing propensity scores (CBPS); the ATT was estimated by full matching with exact matching on key variables, and the ATO by overlap weighting under each specification, with E-values computed for key risk-ratio estimates; and (2) a 2-period difference-in-differences (DID) model with patient fixed effects and cluster-robust standard errors, using pre- vs postoperative hydrocephalus as the time dimension, with robustness examined by wild cluster bootstrap, randomization inference, and placebo DID. Analyses were performed in R 4.4.1 with 2-sided *P *< .05. Detailed specifications, covariate lists, and sensitivity analysis settings are provided in Supplementary Methods S1.19-S1.27.

## Results

### Baseline Characteristics of the Cohort

Among 106 patients with TG, 65 (61.3%) had DMG and 41 (38.7%) non-DMG; 87 (82.1%) were adults and 19 (17.9%) were pediatric. Sex distribution was similar across groups. DMG patients were younger than non-DMG (30.2 ± 14.4 vs 45.1 ± 18.5 years, *P *< .001) and almost all had WHO grade 4 tumors (100% vs 90.2%, *P *= .02). Compared with non-DMG, DMG less often extended into cerebral lobes (3.1% vs 17.1%, *P *= .032), was associated with fewer preoperative motor deficits (hemiparesis 33.8% vs 68.3%, *P *= .001), and slightly higher preoperative KPS (75.2 ± 9.4 vs 68.8 ± 13.8, *P *= .005), whereas rates of hydrocephalus, midbrain or basal ganglia involvement, EOR, and postoperative KPS were similar. As expected, all DMGs were H3K27-altered with loss of H3K27me3, and all non-DMGs were H3K27M-wildtype with retained H3K27me3 (both *P *< .001). MGMT promoter methylation was more frequent in non-DMG (29.3% vs 7.7%, *P *= .002) and strong p53 immunopositivity in DMG (41.5% vs 9.8%, *P *= .006); tumor mutation burden was comparable between groups.

While H3K27-altered DMG were the predominant diagnosis in both pediatric (73.7%) and adult (58.6%) cohorts, the distribution of non-DMG tumors differed significantly by age. Adult non-DMG tumors were exclusively high-grade malignancies (eg, *IDH*-wild-type glioblastoma). In contrast, the pediatric cohort exhibited a more dichotomous histological spectrum; alongside the high prevalence of DMG, pediatric patients had a significantly higher proportion of circumscribed low-grade gliomas (WHO grade 1-2 15.8% vs 0.0%, *P *= .009), lower Ki-67 index (Ki-67 < 5%: 21.1% vs 4.6%, *P *= .027), more frequent transfrontal+mTV procedures (21.1% vs 4.6%, *P *= .042) and higher postoperative KPS (64.7 ± 8.4 vs 57.4 ± 12.8, *P *= .018) than adults, while rates of hydrocephalus, hemiparesis, and anatomical involvement patterns were largely similar. Detailed baseline characteristics are summarized in Table 1.

**Table 1.** Baseline clinical, radiological, and molecular characteristics of the cohort stratified by diagnosis (DMG vs non-DMG) and age group (adult vs pediatric)

Variable	Overall (*n* = 106)	Diagnosis			Age group		
		DMG (*n* = 65)	non-DMG (*n* = 41)	*P*-value	Adult (*n* = 87)	Pediatric (*n* = 19)	*P*-value
**Clinical and demographics**							
Gender, *n* (%)				.776			.521
Female	46 (43.4)	27 (41.5)	19 (46.3)		36 (41.4)	10 (52.6)	
Male	60 (56.6)	38 (58.5)	22 (53.7)		51 (58.6)	9 (47.4)	
Age (years) (mean ± SD)				**<.001**			**<.001**
	35.98 (17.59)	30.23 (14.40)	45.10 (18.48)		41.60 (13.99)	10.26 (4.32)	
WHO grade, *n* (%)				**.02**			**.009**
1	1 (0.9)	0 (0.0)	1 (2.4)		0 (0.0)	1 (5.3)	
2	2 (1.9)	0 (0.0)	2 (4.9)		0 (0.0)	2 (10.5)	
3	1 (0.9)	0 (0.0)	1 (2.4)		1 (1.1)	0 (0.0)	
4	102 (96.2)	65 (100.0)	37 (90.2)		86 (98.9)	16 (84.2)	
Chief complaint, *n* (%)				.145			.524
Dizziness	12 (11.3)	7 (10.8)	5 (12.2)		10 (11.5)	2 (10.5)	
Intracranial hypertension	48 (45.3)	33 (50.8)	15 (36.6)		39 (44.8)	9 (47.4)	
Motor disturbance	21 (19.8)	8 (12.3)	13 (31.7)		15 (17.2)	6 (31.6)	
Sensory dysfunction	11 (10.4)	6 (9.2)	5 (12.2)		11 (12.6)	0 (0.0)	
Visual/ocular disturbances	7 (6.6)	6 (9.2)	1 (2.4)		6 (6.9)	1 (5.3)	
Others	7 (6.6)	5 (7.7)	2 (4.9)		6 (6.9)	1 (5.3)	
**Imaging characteristics**							
Tumor laterality of thalamic origin, *n* (%)				.448			.089
Left	52 (49.1)	32 (49.2)	20 (48.8)		44 (50.6)	8 (42.1)	
Right	53 (50.0)	33 (50.8)	20 (48.8)		43 (49.4)	10 (52.6)	
Bilateral	1 (0.9)	0 (0.0)	1 (2.4)		0 (0.0)	1 (5.3)	
Involvement of the contralateral thalamus, *n* (%)				.15			.745
Yes	17 (16.0)	14 (21.5)	3 (7.3)		15 (17.2)	2 (10.5)	
No	80 (75.5)	46 (70.8)	34 (82.9)		65 (74.7)	15 (78.9)	
Unknown	9 (8.5)	5 (7.7)	4 (9.8)		7 (8.0)	2 (10.5)	
Involvement of the midbrain, *n* (%)				.708			.574
Yes	58 (54.7)	37 (56.9)	21 (51.2)		46 (52.9)	12 (63.2)	
No	48 (45.3)	28 (43.1)	20 (48.8)		41 (47.1)	7 (36.8)	
Involvement of the basal ganglia, *n* (%)				1			1
Yes	21 (19.8)	13 (20.0)	8 (19.5)		17 (19.5)	4 (21.1)	
No	85 (80.2)	52 (80.0)	33 (80.5)		70 (80.5)	15 (78.9)	
Involvement of the cerebral lobe(s), *n* (%)				**.032**			.299
Yes	9 (8.5)	2 (3.1)	7 (17.1)		9 (10.3)	0 (0.0)	
No	96 (90.6)	62 (95.4)	34 (82.9)		77 (88.5)	19 (100.0)	
Unknown	1 (0.9)	1 (1.5)	0 (0.0)		1 (1.1)	0 (0.0)	
Involvement of the lateral ventricular wall, *n* (%)				.116			1
Yes	6 (5.7)	6 (9.2)	0 (0.0)		5 (5.7)	1 (5.3)	
Unknown	100 (94.3)	59 (90.8)	41 (100.0)		82 (94.3)	18 (94.7)	
Maximum tumor diameter, mm (mean ± SD)				.906			.598
	43.52 (10.56)	43.62 (10.35)	43.37 (11.01)		43.26 (10.73)	44.68 (9.94)	
Enhancement, *n* (%)				.59			.541
Homogeneous	8 (7.5)	5 (7.7)	3 (7.3)		8 (9.2)	0 (0.0)	
Partial	76 (71.7)	44 (67.7)	32 (78.0)		61 (70.1)	15 (78.9)	
None	21 (19.8)	15 (23.1)	6 (14.6)		17 (19.5)	4 (21.1)	
Missing	1 (0.9)	1 (1.5)	0 (0.0)		1 (1.1)	0 (0.0)	
Cystic change, *n* (%)				.412			.867
Yes	47 (44.3)	26 (40.0)	21 (51.2)		38 (43.7)	9 (47.4)	
No	58 (54.7)	38 (58.5)	20 (48.8)		48 (55.2)	10 (52.6)	
Missing	1 (0.9)	1 (1.5)	0 (0.0)		1 (1.1)	0 (0.0)	
Peritumoral edema, *n* (%)				.158			.381
Severe	2 (1.9)	0 (0.0)	2 (4.9)		2 (2.3)	0 (0.0)	
Mild	28 (26.4)	16 (24.6)	12 (29.3)		25 (28.7)	3 (15.8)	
None	76 (71.7)	49 (75.4)	27 (65.9)		60 (69.0)	16 (84.2)	
**Treatment and perioperative status**							
Surgical approach, *n* (%)				.666			**.042**
TPO-trigone	59 (55.7)	34 (52.3)	25 (61.0)		51 (58.6)	8 (42.1)	
Transfrontal	39 (36.8)	26 (40.0)	13 (31.7)		32 (36.8)	7 (36.8)	
Transfrontal + ETV	8 (7.5)	5 (7.7)	3 (7.3)		4 (4.6)	4 (21.1)	
EOR, *n* (%)				.547			1
GTR or STR	78 (73.6)	46 (70.8)	32 (78.0)		64 (73.6)	14 (73.7)	
PR	28 (26.4)	19 (29.2)	9 (22.0)		23 (26.4)	5 (26.3)	
Postoperative treatment, *n* (%)				.617			.763
Lost to follow-up	19 (17.9)	14 (21.5)	5 (12.2)		16 (18.4)	3 (15.8)	
No treatment	2 (1.9)	1 (1.5)	1 (2.4)		1 (1.1)	1 (5.3)	
Chemotherapy (temozolomide)	8 (7.5)	4 (6.2)	4 (9.8)		7 (8.0)	1 (5.3)	
Stupp’s regimen	73 (68.9)	44 (67.7)	29 (70.7)		59 (67.8)	14 (73.6)	
Stupp’s regimen + dordaviprone	1 (0.9)	1 (1.5)	0 (0)		1 (1.1)	0 (0)	
Second surgery after recurrence + Stupp’s regimen	3 (2.8)	1 (1.5)	2 (4.9)		3 (3.4)	0 (0)	
**Preoperative hydrocephalus, n (%)**				.11			.495
Yes	80 (75.5)	53 (81.5)	27 (65.9)		64 (73.6)	16 (84.2)	
No	26 (24.5)	12 (18.5)	14 (34.1)		23 (26.4)	3 (15.8)	
Postoperative hydrocephalus, *n* (%)				.906			.373
Yes	46 (43.4)	29 (44.6)	17 (41.5)		40 (46.0)	6 (31.6)	
No	60 (56.6)	36 (55.4)	24 (58.5)		47 (54.0)	13 (68.4)	
Preoperative hemiparesis, *n* (%)				**.001**			.198
Yes	50 (47.2)	22 (33.8)	28 (68.3)		38 (43.7)	12 (63.2)	
No	56 (52.8)	43 (66.2)	13 (31.7)		49 (56.3)	7 (36.8)	
Postoperative hemiparesis, *n* (%)				.136			.558
Yes	81 (76.4)	46 (70.8)	35 (85.4)		65 (74.7)	16 (84.2)	
No	25 (23.6)	19 (29.2)	6 (14.6)		22 (25.3)	3 (15.8)	
Preoperative KPS (mean ± SD)				**.005**			.365
	72.74 (11.67)	75.23 (9.37)	68.78 (13.82)		73.22 (11.66)	70.53 (11.77)	
Postoperative KPS (mean ± SD)				.089			**.018**
	58.68 (12.43)	60.31 (12.74)	56.10 (11.59)		57.36 (12.80)	64.74 (8.41)	
**Molecular markers**							
*IDH* mutation status, *n* (%)				—			—
Yes	0 (0)	0 (0)	0 (0)		0 (0)	0 (0)	
No	106 (100.0)	65 (100.0)	41 (100.0)		87 (100.0)	19 (100.0)	
1p/19q codeletion status, *n* (%)				.405			.848
Codeleted	2 (1.9)	1 (1.5)	1 (2.4)		2 (2.3)	0 (0.0)	
Non-codeleted	77 (72.6)	50 (76.9)	27 (65.9)		62 (71.3)	15 (78.9)	
Unknown	27 (25.5)	14 (21.5)	13 (31.7)		23 (26.4)	4 (21.1)	
H3K27M mutation status, *n* (%)				**<.001**			.301
Positive	65 (61.3)	65 (100.0)	0 (0.0)		51 (58.6)	14 (73.7)	
Negative	41 (38.7)	0 (0.0)	41 (100.0)		36 (41.4)	5 (26.3)	
H3K27me expression status, *n* (%)				**<.001**			.301
Positive	41 (38.7)	0 (0.0)	41 (100.0)		36 (41.4)	5 (26.3)	
Negative	65 (61.3)	65 (100.0)	0 (0.0)		51 (58.6)	14 (73.7)	
MGMT promoter methylation status, *n* (%)				**.002**			.792
Methylated	17 (16.0)	5 (7.7)	12 (29.3)		15 (17.2)	2 (10.5)	
Unmethylated	50 (47.2)	38 (58.5)	12 (29.3)		41 (47.1)	9 (47.4)	
Unknown	39 (36.8)	22 (33.8)	17 (41.5)		31 (35.6)	8 (42.1)	
p53 expression status, *n* (%)				**.006**			.403
Strong positive (++/+++)	31 (29.2)	27 (41.5)	4 (9.8)		24 (27.6)	7 (36.8)	
Weak positive (+)	46 (43.4)	23 (35.4)	23 (56.1)		39 (44.8)	7 (36.8)	
Negative	21 (19.8)	11 (16.9)	10 (24.4)		16 (18.4)	5 (26.3)	
Unclassified	8 (7.5)	4 (6.2)	4 (9.8)		8 (9.2)	0 (0.0)	
Ki-67 labeling index, *n* (%)				.074			**.027**
>20%	78 (73.6)	46 (70.8)	32 (78.0)		64 (73.6)	14 (73.7)	
5-20%	20 (18.9)	16 (24.6)	4 (9.8)		19 (21.8)	1 (5.3)	
<5%	8 (7.5)	3 (4.6)	5 (12.2)		4 (4.6)	4 (21.1)	
TMB, mut/Mb (mean ± SD)				.684			.193
	4.20 (2.56)	4.12 (1.86)	4.33 (3.42)		4.35 (2.60)	3.51 (2.34)	

Values are presented as *n* (%) or mean ± SD. *P*-values refer to comparisons between DMG and non-DMG, and between adult and pediatric patients, respectively. Bold values indicate statistically significant results.

### Genomic Landscape of TGs Across Age and Diagnostic Groups

Across 106 TGs, we observed a stereotyped pattern of somatic alterations (Figure 1). *H3F3A*, *TP53,* and *ATRX* were the most frequently mutated genes, with additional recurrent events in DNA repair/chromatin regulators and RTK/PI3K pathway genes. Copy-number analysis revealed recurrent copy-number gains/amplifications involving *TP53*, *IGF1R*, *EGFR*, *KIT*, and *RICTOR*, and recurrent deletions affecting loci including *CDKN2A/B*, *POLD1*, and *DLL3*, highlighting co-occurrence of *H3F3A*, *TP53*, and *ATRX* mutations with multiple RTK/RAS/PI3K lesions and CNVs events.

**Figure 1.**
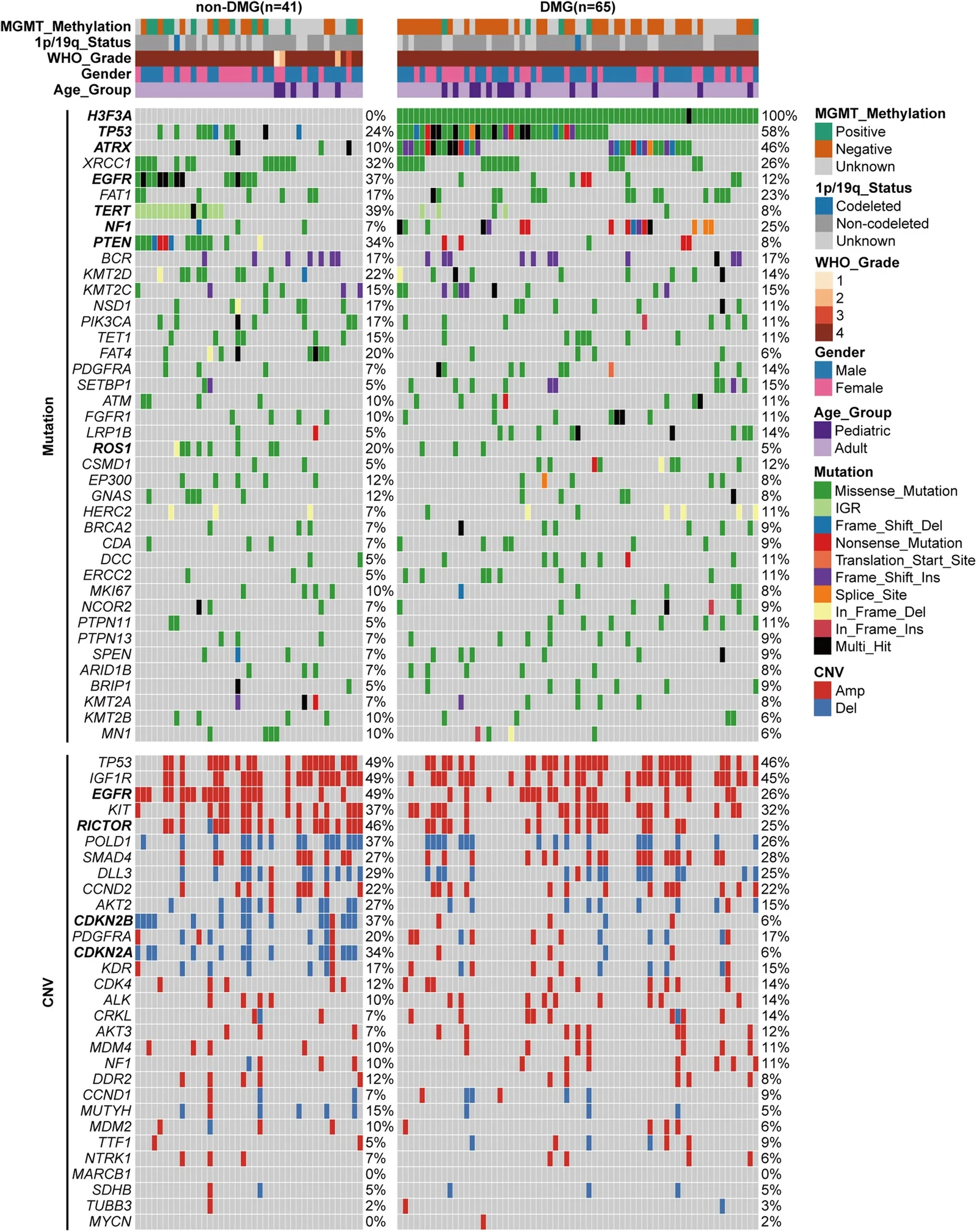
Integrated mutational and copy-number landscape of thalamic gliomas (TGs) stratified by diagnosis. Oncoplot of somatic single-nucleotide variants (SNVs)/indels (top) and copy-number alterations (CNVs; bottom) across 106 TGs, stratified by diagnosis (non-diffuse midline glioma [DMG], *n* = 41; DMG, *n* = 65). Columns represent individual tumors and rows represent selected recurrently altered genes. Mutation classes and CNV states (amplification vs deletion) are color-coded. Annotation tracks summarize MGMT promoter methylation status, 1p/19q codeletion, WHO grade, sex, diagnosis, and age group. Percentages shown beside each gene indicate the corresponding alteration frequencies for SNVs/indels or CNVs. Gene names shown in bold in the gene-label column denote significantly different alteration frequencies between non-DMG and DMG tumors, including *H3F3A*, *TP53*, *ATRX*, *EGFR*, *TERT*, *NF1*, *PTEN*, and *ROS1* in the SNV/indel panel, and *EGFR*, *RICTOR*, *CDKN2B*, and *CDKN2A* in the CNV panel (Fisher’s exact test, *P* < 0.05).

Stratification by diagnostic group (Figure 1) delineated a restricted set of alterations that differed significantly between non-DMG and DMG. *H3F3A* mutations were confined to DMG (non-DMG vs DMG: 0% vs 100%) and were accompanied by higher frequencies of *TP53* (24% vs 58%) and *ATRX* (10% vs 46%) mutations, consistent with the canonical *H3F3A*-TP53-ATRX triad. In contrast, non-DMG tumors more frequently harbored mutations in *EGFR* (37% vs 12%), *TERT* (39% vs 8%), *PTEN* (34% vs 8%), and *ROS1* (20% vs 5%), whereas *NF1* mutations were enriched in DMG (7% vs 25%). At the copy-number level, non-DMG exhibited higher rates of predominantly amplificatory events involving *EGFR* (49% vs 26%) and *RICTOR* (46% vs 25%), as well as predominantly deletions affecting *CDKN2A* (34% vs 6%) and *CDKN2B* (37% vs 6%). All DMGs harbored canonical *H3F3A* K27M mutations; no H3-wildtype *EGFR*-driven DMG phenotype or *EZHIP* sequence alteration was detected by targeted sequencing.

Stratification by age (Figure S1) identified comparatively fewer significant differences: pediatric tumors showed higher mutation rates in *LRP1B* (26% vs 7%) and *MN1* (26% vs 3%), whereas adult tumors were enriched for CNVs, including *CCND2* amplification (0% vs 26%) and deletions of *CDKN2B* (0% vs 22%) and *CDKN2A* (0% vs 21%) (pediatric vs adult).

*BRAF* alterations were infrequent and heterogeneous, comprising 3 canonical V600E mutations, 3 non-hotspot missense variants, and several intronic-only variants; no recurrent *BRAF*-defined clinicomolecular subgroup was identified (Table S1).

### Mutual Exclusivity Between H3F3A Mutation and 9p21 Loss

We examined the interplay between *H3F3A* mutation, *CDKN2A/B* (9p21) deletion, and RTK/PI3K pathway alterations. A pairwise enrichment map integrating SNVs, indels, and CNVs showed structured co-occurrence and depletion patterns (Figure 2A): *H3F3A* mutation was markedly depleted in tumors with 9p21 loss, whereas 9p21 loss preferentially co-occurred with RTK/PI3K lesions such as *EGFR* amplification. In alluvial plots, most DMG cases aligned along an “H3K27 axis,” while a substantial subset of non-DMG gliomas followed a “9p21/cell-cycle axis” frequently combined with RTK/PI3K alterations (Figure 2B).

**Figure 2.**
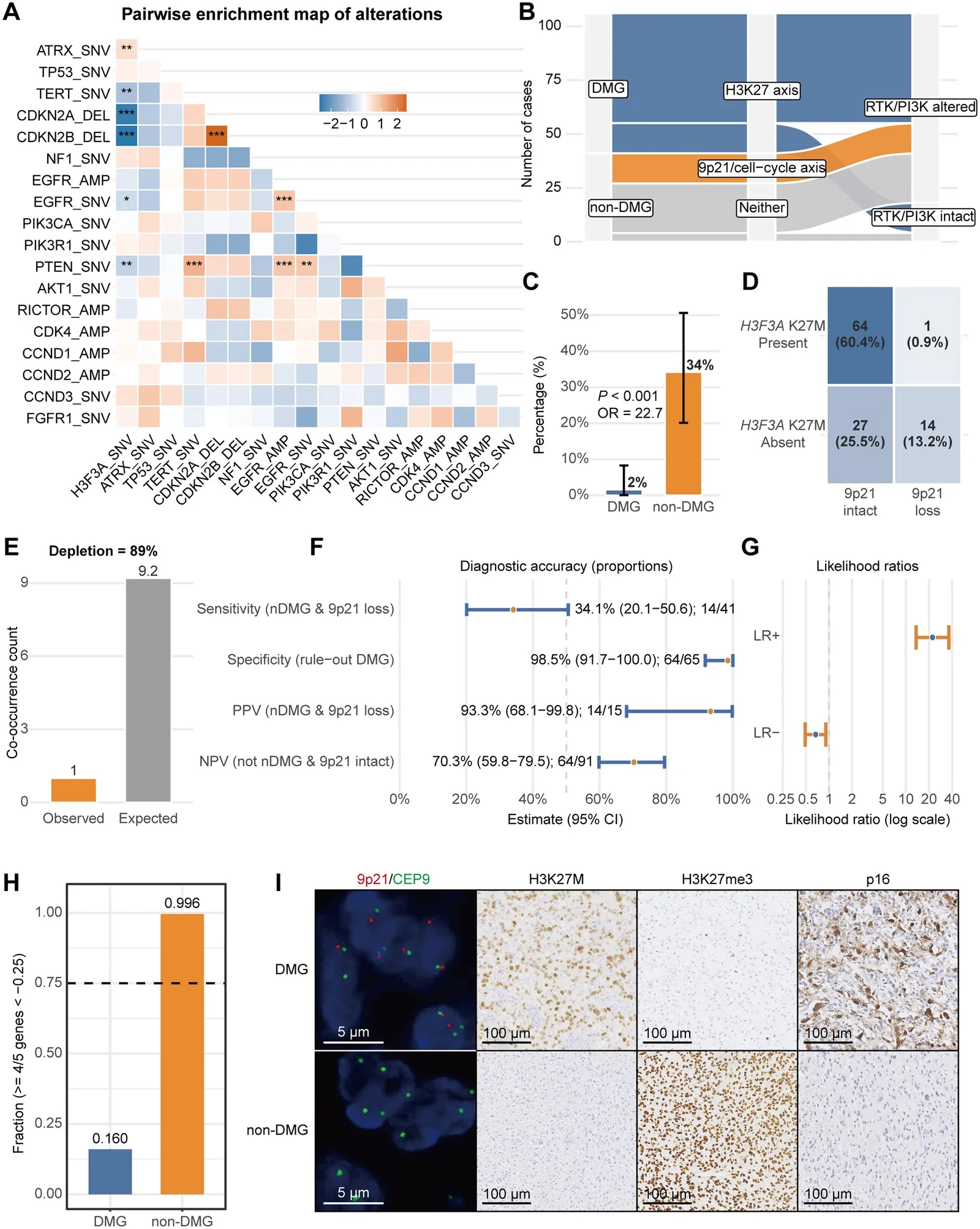
Near mutual exclusivity between *H3F3A* mutation and *CDKN2A/B* (9p21) loss and multilevel validation. (A) Pairwise enrichment map of selected single-nucleotide variants (SNVs)/indels and copy-number alteration (CNV) events. Tiles show log₂(observed/expected) co-occurrence under independence; negative values indicate depletion/exclusivity, whereas positive values indicate enrichment/co-occurrence; asterisks indicate false discovery rate (FDR)–adjusted *P* < .05 (Fisher’s exact test). (B) Alluvial plot showing flows from diagnosis (diffuse midline glioma [DMG] vs non-DMG) to driver axis assignment (H3K27 axis, 9p21/cell-cycle axis, or neither) and RTK/PI3K pathway status (altered vs intact); bar heights denote case counts. (C) Frequency of 9p21 loss (*CDKN2A/B* deletion) by diagnosis with 95% binomial CIs; Fisher’s exact P, odds ratio, and risk difference are reported in the panel subtitle. (D) Joint distribution of *H3F3A* K27M mutation and 9p21 loss (counts and percentages). (E) Observed versus expected number of tumors with both *H3F3A* K27M mutation and 9p21 loss under an independence assumption; depletion is annotated. (F) Diagnostic accuracy of 9p21 loss for identifying non-DMG (sensitivity, specificity, positive predictive value [PPV], negative predictive value [NPV]) with 95% CIs. (G) Positive (LR⁺) and negative (LR⁻) likelihood ratios for 9p21 loss (log scale). (H) Single-cell core-gene classifier summary based on the proportion of malignant cells in which at least 4 of 5 core 9p21 genes showed down-shifted expression; dashed lines indicate prespecified decision thresholds. (I) Orthogonal tissue-based validation by interphase FISH and IHC. FISH was performed using probes targeting 9p21 and CEP9. Representative IHC images show H3K27M, H3K27me3 and p16 staining in DMG and non-DMG cases. p16 IHC is shown as a complementary protein-level correlate and was not used to define or substitute for homozygous CDKN2A/B loss.

*CDKN2A/B* loss occurred in 34.1% of non-DMG tumors (14/41) but only 1.5% of DMG (1/65; Figure 2C), yielding an odds ratio of 22.7 for non-DMG versus DMG (Fisher’s exact *P *= 3.6 × 10⁻⁶). Only 1 tumor in the cohort carried both *H3F3A* mutation and 9p21 loss, compared with 9.2 expected under independence, corresponding to ∼89% depletion of co-occurrence (Figure 2D and E).

Using non-DMG as the target condition and 9p21 loss as the test, sensitivity was 34.1%, and specificity for ruling out DMG was 98.5% (Figure 2F). The PPV and NPV for non-DMG were 93.3% and 70.3%, with a positive likelihood ratio of 22.2 and a negative likelihood ratio of 0.67 (Figure 2G). Thus, 9p21 loss is relatively insensitive but highly specific for identifying non-DMG and effectively rules out DMG in this setting.

To examine 9p21 status at single-cell resolution, we performed droplet-based scRNA-seq on 1 H3K27-altered DMG and 1 non-DMG tumor. After integration, malignant and non-malignant compartments were separated by CNV burden, with broadly similar immune and stromal compositions between tumors (Figure S2A-H). Focusing on malignant clusters, we derived 4 expression-based metrics of 9p21.3 status. In the window around chr9:21-23 Mb (Figure S2I), only a minority of DMG malignant cells fell below the Δlog₂ threshold, whereas the non-DMG tumor showed a broad loss-like shift. Within the core window containing *MTAP*, *CDKN2A*, *CDKN2B-AS1,* and *CDKN2B* (Figure S2J), the non-DMG sample exhibited coordinated down-regulation of all genes, while DMG cells remained close to baseline. Run-length statistics (Figure S2K) showed only short loss-like segments in scattered DMG cells but long runs of consecutive low-expression genes in most non-DMG malignant cells. The core-4 classifier (≥4/5 core genes down-shifted; Figure 2H) accordingly labeled only about one-tenth of DMG malignant cells as “9p21-loss-like” versus almost all non-DMG malignant cells.

Interphase FISH showed a preserved 9p21/CEP9 ratio in DMG (>0.86) and a markedly reduced ratio in non-DMG (<0.50), and IHC showed H3K27M positivity with retained p16 in DMG versus H3K27M negativity with loss of p16 in non-DMG (Figure 2I). Together, these data support 9p21-region loss as a recurrent feature of the non-DMG molecular axis and reinforce its near mutual exclusivity with *H3F3A* mutation.

### mTV Is Associated With a Reduced Risk of Postoperative Hydrocephalus

Among 106 patients, 75.5% (80/106) had preoperative hydrocephalus and 43.4% (46/106) had postoperative hydrocephalus, an absolute reduction of ∼32 percentage points after tumor surgery and CSF-diverting procedures (Figure 3A).

**Figure 3.**
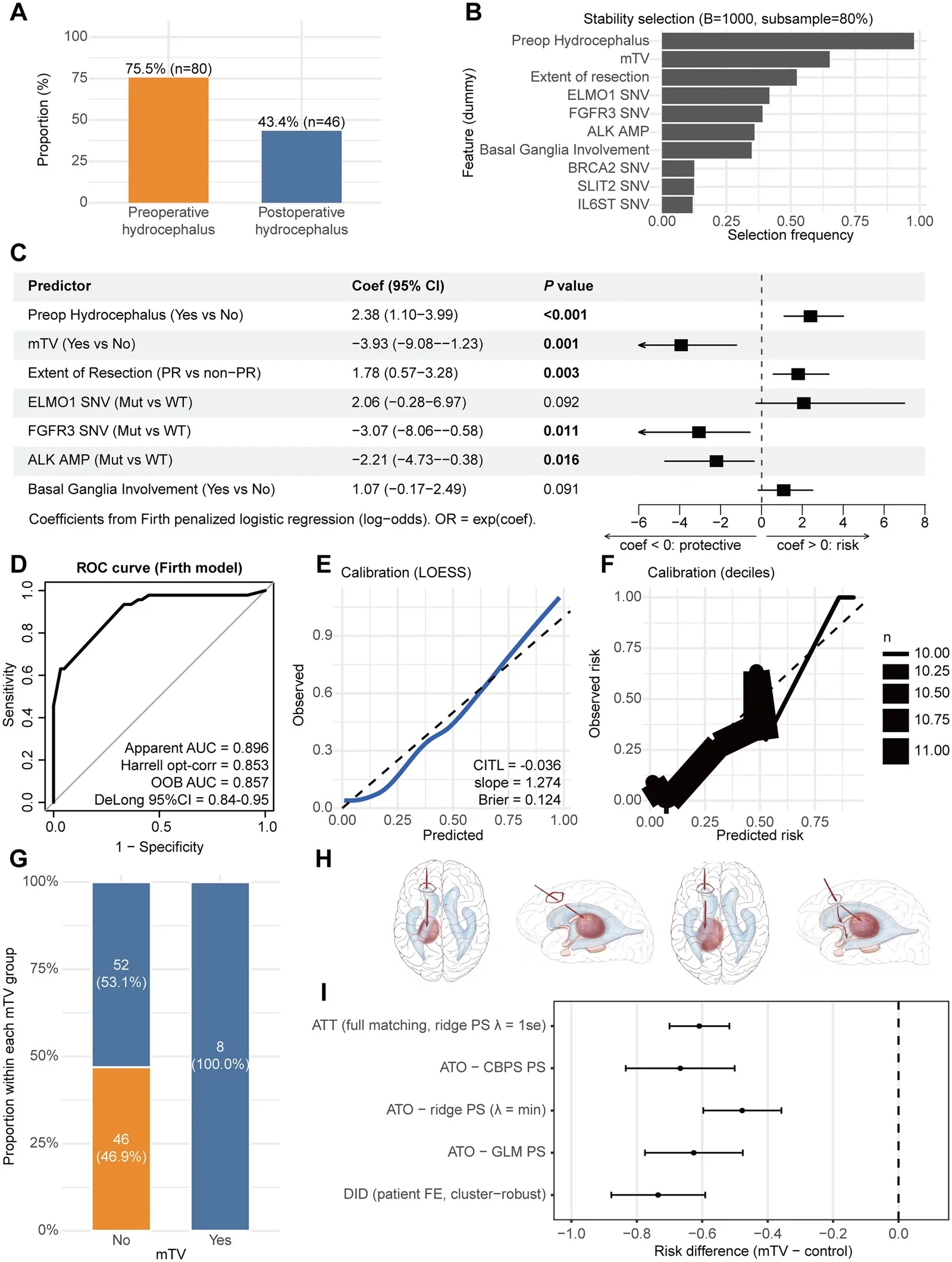
Protective association of mTV with postoperative hydrocephalus. (A) Bar plot comparing the overall prevalence of hydrocephalus before and after tumor surgery in the cohort. (B) Stability-selection plot from LASSO logistic regression showing selection frequencies of candidate predictors across bootstrap subsamples. (C) Forest plot of coefficients from the Firth penalized logistic regression model, with 95% CIs; coefficients left of the vertical line indicate a protective association and those to the right indicate increased risk of postoperative hydrocephalus. (D) ROC curve of the Firth model illustrating overall discrimination. (E) LOESS-smoothed calibration curve comparing predicted and observed risks. (F) Decile-based calibration plot showing observed risk within deciles of predicted probability; point size reflects the number of patients per decile. (G) Stacked bar chart of postoperative hydrocephalus stratified by mTV status, displaying the proportions of patients with and without postoperative hydrocephalus within each mTV group. (H) Schematic illustration of the surgical approaches and the use of microsurgical third ventriculostomy in the management strategy for TGs. (I) Summary forest plot of absolute risk differences for postoperative hydrocephalus comparing mTV with non-mTV management across different causal estimands (ATT, ATO based on overlap weights with alternative propensity score specifications, and DID). Abbreviations: LASSO, least absolute shrinkage and selection operator; LOESS, locally estimated scatterplot smoothing.

LASSO-based stability selection identified preoperative hydrocephalus, mTV, EOR, basal ganglia involvement, and 3 genomic alterations (*ELMO1* SNV, *FGFR3* SNV, and *ALK* amplification) as the most frequently selected predictors of postoperative hydrocephalus; most demographic and radiological covariates were rarely selected (Figure 3B). A multivariable Firth penalized logistic regression including these variables (Figure 3C) showed strong associations of preoperative hydrocephalus (coefficient 2.38, 95% CI 1.10-3.99; *P *< .001) and partial resection (1.78, 0.57-3.28; *P *= .003) with increased log-odds of postoperative hydrocephalus. mTV had a large negative coefficient (−3.93, −9.08 to −1.23; *P *= .001), indicating a pronounced protective association. *FGFR3* SNVs and *ALK* amplification were likewise protective (−3.07, −8.06 to −0.58; *P *= .011 and −2.21, −4.73 to −0.38; *P *= .016), whereas *ELMO1* SNVs and basal ganglia involvement showed positive but less precise effects (*P *= .092 and *P *= .091). Model performance was good (apparent AUC 0.896; optimism-corrected AUC 0.853; out-of-bag AUC 0.857; DeLong 95% CI 0.84-0.95) with acceptable calibration (CITL −0.036, slope 1.274, Brier 0.124; Figure 3D-F).

Because mTV was the strongest modifiable predictor, we examined its effect more closely. In the unmatched data, postoperative hydrocephalus occurred in 46.9% of patients without mTV (46/98) but in 0/8 patients with mTV (Figure 3G); Figure 3H schematizes the surgical strategies. For the ATT estimand, propensity-score full matching with ridge-penalized scores and exact matching on preoperative hydrocephalus and approach yielded an unadjusted risk difference of −0.582 (95% CI −1.011 to −0.153; *P *= .016) and an adjusted RD_ms of −0.609 (95% CI −0.700 to −0.517; bootstrap 95% CI −0.870 to −0.000), corresponding to a 60.9-percentage-point absolute risk reduction in the treated group. The matched Firth odds ratio was 0.01 (95% CI 0.00-0.24; *P *= .0016), with a Haldane risk ratio of 0.096 and a protective E-value of 20.34 (Table S2).

Across 9 ATT sensitivity specifications (Table S3), estimates remained consistently protective (RD_ms −0.381 to −0.800). Firth ORs ranged from 0.01 to 0.15 (significant in 6/9 specifications), while the remaining specifications showed concordant directionality but reduced precision; Haldane RRs were 0.082-0.357 and protective E-values 5.04-23.84.

Overlap-weighted ATO analyses gave concordant evidence (Table S4). Using CBPS-based, ridge-penalized and logistic-regression propensity scores, RD_ms estimates were −0.667 (95% CI −0.834 to −0.501), −0.478 (−0.597 to −0.358), and −0.626 (−0.775 to −0.476), respectively. In all 3 weighted samples, the estimated risk in the mTV group was essentially zero, Firth odds ratios were close to 0 with very small *P*-values, Haldane risk ratios were ≈0.01-0.02, and protective E-values exceeded 100.

DID analyses using patient fixed effects, with preoperative hydrocephalus as baseline and postoperative hydrocephalus as follow-up, further supported a strong protective effect (Table S5). The main DID model estimated a reduction of −0.735 in hydrocephalus probability associated with mTV (95% CI −0.878 to −0.591; *P* ≈ 5 × 10⁻¹⁷). Wild-cluster bootstrap CIs, restriction to same-approach patients, leave-one-out exclusion of each mTV patient, and randomization-inference tests all yielded Δ estimates around −0.70 to −0.74 with highly significant *P*-values, whereas a placebo DID restricted to controls produced a mean Δ of −0.110 and empirical *P *≈ 1.00.

Across penalized regression, propensity-score analyses (ATT/ATO) and DID (Figure 3I; Tables S2-S5), effect estimates were large and directionally consistent, supporting a robust protective association between mTV and postoperative hydrocephalus. Figure S3 illustrates this at the patient level: in a case treated without mTV, persistent postoperative ventriculomegaly necessitated ventriculo-peritoneal shunting despite good tumor control, whereas in a similar case treated with combined resection and mTV, ventricular enlargement and periventricular edema resolved without shunt insertion, and follow-up MRI showed durable tumor control with restored aqueductal patency.

### Predictors of Postoperative Motor Deficits

All 106 patients with complete perioperative motor assessments were included. Both pre- and postoperative hemiparesis were frequent, with only a modest change in overall prevalence (Figure 4A).

**Figure 4.**
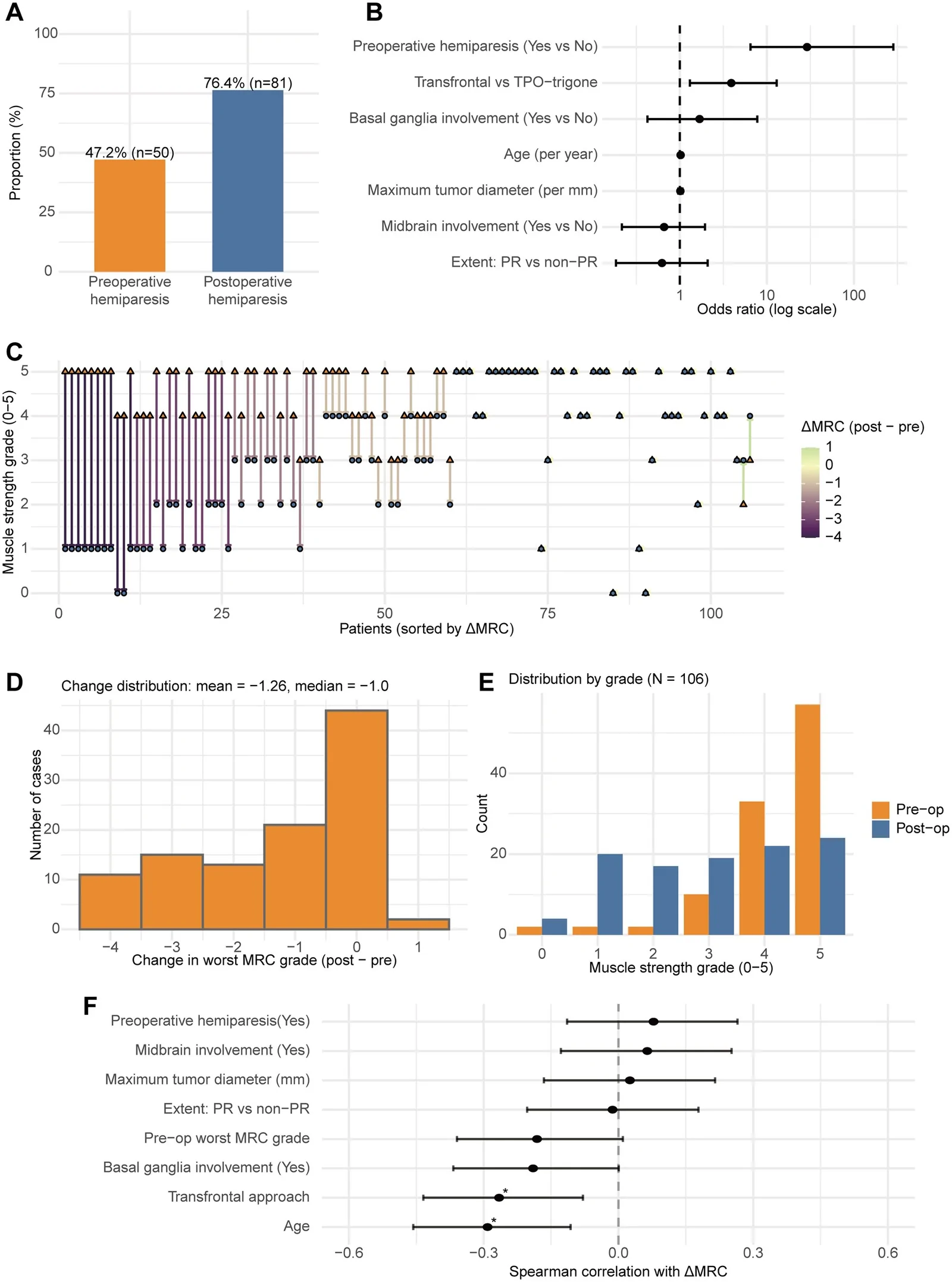
Perioperative motor deficits and their determinants. (A) Bar plot comparing the proportion of patients with hemiparesis before and after surgery. (B) Forest plot of Firth penalized logistic regression showing adjusted odds ratios and 95% CIs for postoperative hemiparesis according to preoperative hemiparesis, age, basal ganglia and midbrain involvement, maximum tumor diameter, extent of resection (partial resection [PR] vs non-PR) and surgical approach (transfrontal vs TPO-trigone). (C) Patient-level trajectories of worst-side Medical Research Council (MRC) grade from pre- to postoperative assessments, with arrows indicating the direction and magnitude of change. (D) Histogram of the change in worst-side MRC grade (ΔMRC = postoperative minus preoperative), illustrating the distribution of motor improvement and deterioration. (E) Side-by-side bar plot of the distribution of worst-side MRC grades (0-5) at the preoperative and postoperative time points. (F) Forest plot of Spearman correlation coefficients between ΔMRC and clinical or surgical covariates, with 95% CIs and asterisk markers for variables that remain significant after false discovery rate correction.

A multivariable Firth penalized logistic regression including preoperative hemiparesis, age, basal ganglia and midbrain involvement, maximum tumor diameter, EOR, and surgical approach (Figure 4B) showed preoperative hemiparesis as the dominant predictor of postoperative hemiparesis (adjusted OR 28.98, 95% CI 6.48-284.05; *P *< .001), indicating an almost 30-fold higher risk in patients with baseline hemiparesis. A transfrontal approach was independently associated with increased risk compared with the TPO-trigone route (adjusted OR 3.90, 95% CI 1.31-12.98; *P *= .014). Age, basal ganglia and midbrain involvement, maximum tumor diameter, and EOR showed no statistically significant independent effects.

At the individual level, trajectories of worst-side MRC grade from pre- to postoperative assessment were heterogeneous (Figure 4C), and the distribution of ΔMRC (postoperative minus preoperative) contained both negative and positive values (Figure 4D). Worst-side MRC grades at each time point spanned the full 0-5 range (Figure 4E), reflecting wide variability in baseline and postoperative limb strength.

In Spearman correlation analyses of continuous predictors of ΔMRC with false discovery rate (FDR) correction (Figure 4F), older age correlated with a more negative ΔMRC (*r* = −0.29, *P *= .002, *q* = 0.020), and a transfrontal approach also correlated with worse ΔMRC (*r* = −0.27, *P *= .006, *q* = 0.024). Basal ganglia involvement and lower preoperative worst-side MRC grade showed borderline trends toward more negative ΔMRC (*r* = −0.19 and −0.18; raw *P *= .05-.06), whereas preoperative hemiparesis, midbrain involvement, maximum tumor diameter, and EOR showed no clear correlations (all q ≥ 0.13). Overall, these findings indicate that pre-existing motor deficit and the choice of a transfrontal approach are the main drivers of postoperative hemiparesis in this cohort.

### OS According to Clinical and Molecular Features

Kaplan-Meier curves for major clinical variables (Figure 5A-L) and selected molecular alterations (Figure S4A-D) showed no significant OS differences between DMG and non-DMG tumors, pediatric and adult patients, or MGMT promoter-methylated versus unmethylated cases, nor according to pre-/postoperative hydrocephalus, pre-/postoperative hemiparesis, TP53 or TERT mutation status, or CDKN2A/B deletion status (all log-rank *P *> .2). In contrast, OS curves separated clearly by EOR (PR vs GTR/STR), TMB (high vs low), and postoperative, but not preoperative, KPS, whereas surgical approach was not associated with OS (Figure 5C-F, K, and L).

**Figure 5.**
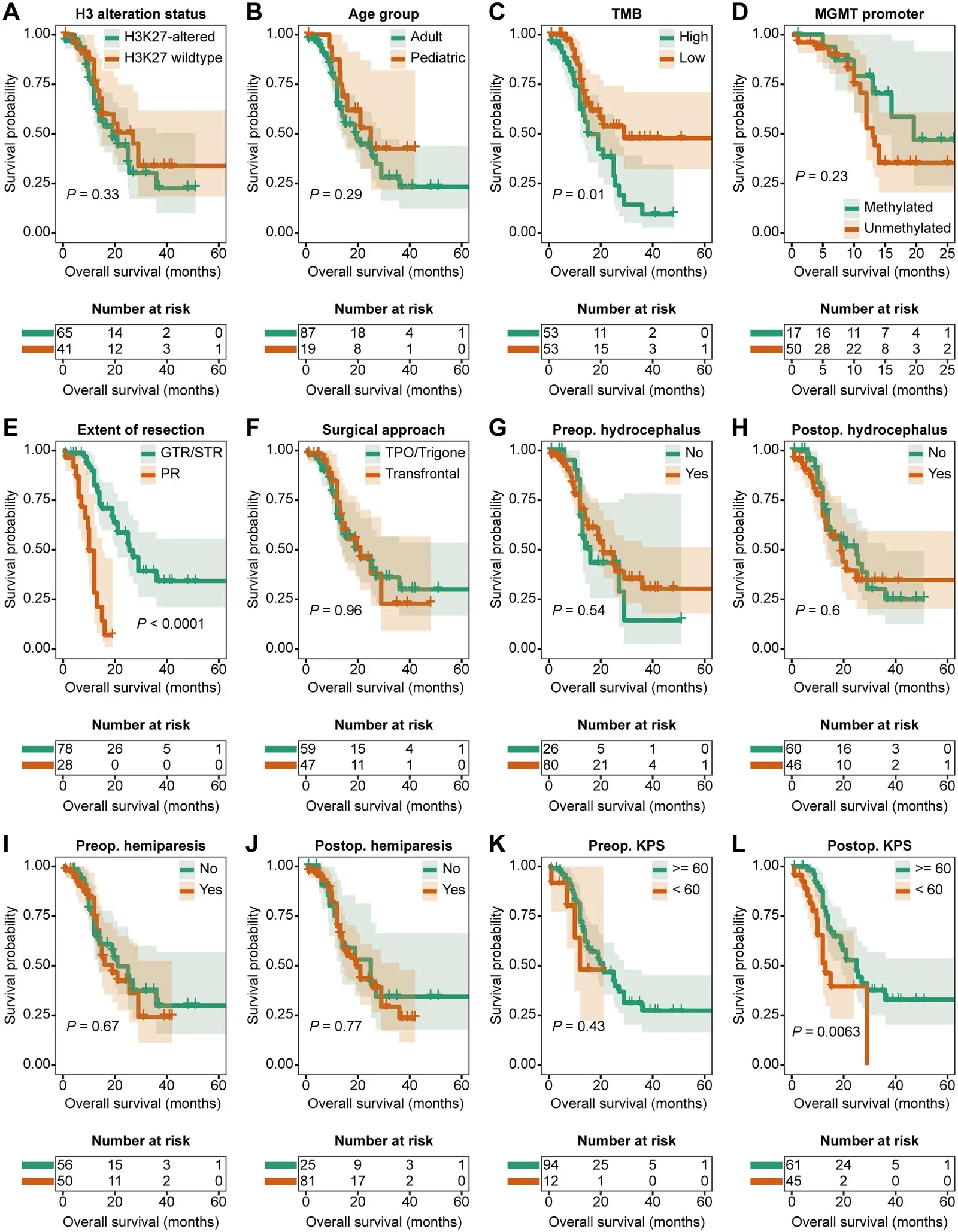
Kaplan-Meier analyses of overall survival in key clinical and molecular subgroups. (A) H3K27-altered vs H3K27 wild-type tumors. (B) Age group (adult vs pediatric). (C) Tumor mutational burden (TMB), dichotomized as low vs high (median split). (D) MGMT promoter methylation status (methylated vs unmethylated). (E) Extent of resection (gross-total/subtotal resection [GTR/STR] vs partial resection [PR]). (F) Surgical approach (TPO-trigone vs transfrontal). (G) Presence versus absence of preoperative hydrocephalus. (H) Presence versus absence of postoperative hydrocephalus. (I) Preoperative hemiparesis (yes vs no). (J) Postoperative hemiparesis (yes vs no). (K) Preoperative KPS (<60 vs ≥60). (L) Postoperative KPS (<60 vs ≥60). For each panel, Kaplan-Meier curves display overall survival in months with corresponding log-rank *P*-values and numbers at risk shown below the *x*-axis.

To quantify these effects, EOR, TMB, postoperative KPS, and DMG status were entered into a multivariable Cox model (Table S6). Partial resection remained a strong independent predictor of worse OS compared with GTR/STR (HR = 5.88, 95% CI 2.81-12.30; *P *< .001). High TMB was also independently associated with a higher hazard of death (HR = 2.02, 95% CI 1.06-3.87; *P *= .033). However, after adjustment, postoperative KPS < 60 (HR = 1.73, 95% CI 0.81-3.68; *P *= .158) and DMG status (HR = 1.24, 95% CI 0.65-2.39; *P *= .513) were not independently associated with OS. Overall, EOR and global mutational burden, rather than postoperative functional status, DMG designation, or individual driver mutations, emerged as the principal independent determinants of survival in this cohort. We further performed stratified prognostic analyses within the DMG and non-DMG cohorts; these analyses showed that EOR was significantly associated with survival in both groups. TMB showed a borderline trend only in the DMG cohort, whereas postoperative KPS was significant only in non-DMG cohort (Table S7).

## Discussion

TGs remain among the most difficult intracranial tumors to treat because of their deep location and proximity to the ventricular system, internal capsule, basal ganglia, and brainstem, with frequent obstructive hydrocephalus and motor pathway involvement.^9-12^ Surgical planning must therefore balance the survival benefit of cytoreduction against the risk of new, potentially permanent neurological deficits.^13^ The literature on TGs remains limited, largely single-institution and retrospective, and has historically been biopsy-dominant. This has limited efforts to link clinical presentation with molecular subtype, perioperative complications, and long-term outcomes.^3^^,^^4^^,^^13^^,^^14^ In this context, our single-center cohort of 106 resected TGs, spanning pediatric and adult patients and including both H3K27-altered DMG and non-DMG entities, enables integrated analysis of clinicoradiological features, molecular profiles, perioperative morbidity, and outcomes.

In our cohort, TGs most commonly presented with manifestations of intracranial hypertension and corticospinal dysfunction. Preoperative hydrocephalus was present in approximately three quarters of patients, and nearly half had preoperative hemiparesis. These features occurred in both age groups, and the frequencies of hydrocephalus and hemiparesis were broadly similar in children and adults. Tumor topography highlighted the thalamus as an anatomic crossroads at the interface of the third ventricle, lateral ventricles, and basal ganglia, with frequent periventricular and midbrain extension and common encroachment on the aqueduct and/or third ventricular floor.^4^^,^^15^^,^^16^

Together, these baseline relationships help explain the frequent coexistence of mass effect, CSF pathway obstruction, and corticospinal tract compromise in TG and the high perioperative risk even before operative manipulation.^13^^,^^14^^,^^16^ From a molecular standpoint, this study adds resection-based genomic data at a scale that is uncommon for TGs. *H3F3A* (H3K27M) alterations were present in roughly 60% of tumors across both pediatric and adult patients, supporting the concept that thalamic DMG is not restricted to childhood in the way diffuse intrinsic pontine glioma is. MGMT promoter methylation was more frequent in non-DMG than in DMG, and *H3F3A* (H3K27M) alterations frequently co-occur with canonical partners such as *TP53* and *ATRX*, consistent with integrated genomic analyses of pediatric and midline high-grade gliomas.^17-19^ These findings reinforce that the thalamus is a major hub for DMG biology while also harboring a substantial subset of H3K27-wildtype high-grade tumors with distinct molecular backgrounds.

Our data show that *CDKN2A/B* (9p21) loss is strongly enriched in non-DMG and rare in DMG within this purely thalamic cohort, reinforcing its near-mutual exclusivity with *H3F3A* alteration. Although this association has been described previously in broader diffuse glioma series,^20^ our study extends its relevance to a large, homogeneous, surgically treated thalamic cohort and demonstrates concordance across targeted sequencing, single-cell reference analysis, FISH, and p16 IHC. In this context, 9p21 loss should not be viewed as a standalone defining feature of non-DMG but rather as a highly specific adjunctive marker favoring non-DMG and arguing against DMG when interpreted alongside H3 alteration status and the overall molecular profile. Because its sensitivity for non-DMG remains limited, absence of 9p21 loss should not be used to diagnose DMG; H3 alteration testing and, when feasible, methylation-based classification should remain the standard.^21^^,^^22^ It is clinically critical to strictly distinguish between homozygous deletion and hemizygous loss of *CDKN2A/B*. As highlighted in the study by Ippen et al accurately distinguishing these states is essential for prognostication, as hemizygous loss does not confer the same adverse survival impact as homozygous deletion and can be susceptible to misinterpretation by algorithmic cutoffs.^23^ In our cohort, the near-mutual exclusivity with H3K27 alterations was mainly driven by homozygous deletions, supporting its role as a highly specific adjunctive marker favoring non-DMG biology. This distinction underscores the real-world utility of FISH. While NGS provides comprehensive profiling, current guidelines (such as EANO and cIMPACT-NOW) emphasize the importance of precise copy number assessment.^24^^,^^25^ FISH serves as a robust orthogonal tool that enables pathologists to directly visualize signal counts, reliably discriminating between biallelic (homozygous) deletion and monoallelic (hemizygous) loss. This makes FISH a practical and accessible method to rule out DMG status with high specificity in clinical settings where NGS may be unavailable or inconclusive. It should be noted that p16 IHC is supportive/illustrative only. DNA methylation profiling provides important support for CNS tumor classification but was not performed in the present study, which should be acknowledged as a limitation; future work will incorporate methylation-based classification, particularly for tumors harboring H3K27 alteration and/or 9p21 loss, to further refine molecular stratification.

Hydrocephalus was the dominant perioperative issue. Preoperatively, 75.5% of patients had ventricular enlargement consistent with obstructive hydrocephalus, and 43.4% developed postoperative hydrocephalus despite resection. Using stability selection with LASSO logistic regression and a parsimonious Firth penalized logistic model, we identified preoperative hydrocephalus, EOR, mTV, basal ganglia involvement, and a small number of molecular features as the most stable predictors of postoperative hydrocephalus. In the final model, preoperative hydrocephalus and PR were strongly associated with increased postoperative hydrocephalus risk, whereas mTV was the most influential modifiable protective factor. Discrimination was strong (apparent AUC 0.896; optimism-corrected AUC 0.853; out-of-bag AUC 0.857; DeLong 95% CI 0.84-0.95), and calibration showed good agreement between predicted and observed risk. Practically, these results indicate that postoperative hydrocephalus risk in TGs can be stratified using variables that are routinely available in clinical care.

Because mTV consistently emerged as a key covariate, we subsequently analyzed mTV as the exposure and evaluated its association with postoperative hydrocephalus using complementary causal-inference approaches. In propensity-score full matching (estimating the ATT), mTV was associated with an absolute reduction in postoperative hydrocephalus risk of approximately 60 percentage points compared with matched non-mTV patients. Overlap weighting (estimating the ATO) across multiple propensity-score specifications yielded concordant effects, with risk reductions typically in the range of ∼0.5-0.7. A DID analysis leveraging paired pre- and postoperative hydrocephalus status supported a similarly large protective association. The concordance in direction and magnitude across methods argues against a model-dependent finding and supports a robust association between mTV and a lower risk of postoperative hydrocephalus. These findings may inform perioperative CSF management. Among patients undergoing a transfrontal tumor resection without mTV, postoperative hydrocephalus was frequent, and a substantial proportion ultimately required ventriculoperitoneal shunting, with ongoing device dependence and risks of infection or malfunction. In contrast, when maximal safe resection was performed with concomitant mTV in anatomically suitable cases, postoperative hydrocephalus was less frequent and the need for permanent CSF diversion was reduced. Taken together, our data suggest that an upfront, single-stage approach, resection with concurrent mTV, should be considered when the third ventricular floor/aqueductal axis is involved and anatomy permits, particularly in patients presenting with preoperative hydrocephalus who are at increased baseline risk of postoperative CSF-related complications. Phase-contrast cine MRI (mid-aqueduct) provided mechanistic context. Preoperatively, CSF flow showed irregular, nonperiodic velocity excursions, consistent with focal aqueductal narrowing and disturbed hydrodynamics. After gross resection with postresection mTV, imaging confirmed aqueductal decompression and restored patency, yet cine flow remained atypical, suggesting persistent alterations in intracranial compliance after chronic obstruction. These findings are compatible with 2 contributors to postoperative hydrocephalus: (1) residual/recurrent outflow obstruction (residual tumor or scarring) and (2) longer-term impairment of CSF absorption and brain/ventricular compliance from prolonged intracranial hypertension. This may explain the prognostic impact of preoperative hydrocephalus and supports considering mTV in selected patients. However, the sample size of patients undergoing synchronous mTV was small (*n* = 8). Although complementary causal-inference models consistently demonstrated a marked reduction in postoperative hydrocephalus, we acknowledge that this represents a preliminary proof-of-concept experience. Given the rarity of TG and the even smaller subset of patients eligible for open resection with synchronous ventriculostomy, future prospective multicenter studies are needed to validate mTV as a broader preventive strategy against hydrocephalus-related morbidity.

Motor function represented a second major contributor to postoperative morbidity. In our cohort, 47.2% of patients had preoperative hemiparesis, and overall strength shifted toward lower MRC grades postoperatively, with a median decline of about 1 grade in the worst-affected limb and a negative mean change in the worst MRC. Children tended to have higher postoperative KPS than adults, consistent with greater neuroplasticity, but early postoperative weakness remained frequent. In multivariable Firth regression, preoperative hemiparesis was the strongest predictor of postoperative motor deterioration, underscoring that once corticospinal fibers have been infiltrated or injured, they are highly vulnerable to additional operative stress. Objective baseline motor assessment, supplemented by tractography when feasible, should therefore be central to counseling and to individualized decisions about resection aggressiveness.

Surgical corridor selection was also associated with motor outcome. Across analyses of MRC change and postoperative hemiparesis, a transfrontal approach was linked to a higher likelihood of motor deterioration than a temporoparieto-occipital transventricular trans-trigonal route, including in adjusted models. This likely reflects both anatomy and selection: with a transventricular trans-trigonal corridor, the operative trajectory generally confers a lower risk of injuring the anterolateral internal capsule and the deep-seated cerebral peduncle, whereas a transfrontal transventricular route carries a relatively higher risk of violating the cerebral peduncle and anterolateral corticospinal fibers. Accordingly, rigorous preoperative assessment and selection of the surgical corridor, together with intraoperative tractography-guided navigation, may help mitigate motor disability while enabling maximal safe cytoreduction. Notably, neuronavigation accuracy can be limited by intraoperative CSF loss and the resulting brain shift; however, because the thalamus is a midline structure, CSF-related target shift may be less pronounced for TGs than for hemispheric tumors.

Although biopsy remains the standard for classic DIPG, unilateral TGs represent a distinct surgical entity. Recent studies suggest that greater EOR may improve survival in selected TGs, although evidence specific to thalamic H3K27-altered DMG remains limited and heterogeneous. Palmisciano et al showed that complete resection was associated with longer survival in adult TGs,^3^ and Martínez-Santos et al reported similar findings in a modern single-center series.^4^ In our homogeneous cohort of 106 pure TGs, including 65 DMG, greater resection remained independently associated with improved OS, whereas H3K27-altered status did not. This is consistent with the 2025 EANS-EANO guidelines supporting maximal safe cytoreduction.^26^ Despite these functional risks, EOR remained a key driver of survival. When dichotomized as “GTR or STR” versus “PR” multivariable Cox models showed that PR carried an approximately 6-fold higher hazard of death after adjustment for age group, TMB group, postoperative performance status, and DMG status. Median OS was 21.0 months (95% CI 15.0-29.0). This compares favorably with historical biopsy-dominant series and is consistent with contemporary surgical reports suggesting improved outcomes with greater resection in TGs.^4^^,^^13^^,^^14^^,^^26^

The absence of an independent prognostic effect of H3K27 alteration in our cohort likely reflects the specific composition of the comparator group and the nature of this resection-based series. Non-DMG were also predominantly highly aggressive WHO grade 4 tumors rather than biologically favorable controls. In addition, EOR and postoperative functional status emerged as stronger determinants of survival, which may have attenuated survival differences attributable to molecular subgroup alone. Nevertheless, larger multicenter studies are needed to further clarify the independent prognostic value of H3K27 status. Coupled with the survival association observed for GTR/STR, these data argue for a pragmatic multimodal strategy: when anatomically and functionally feasible, aim for maximal safe cytoreduction to reduce tumor burden and obtain adequate tissue for comprehensive molecular profiling, and then facilitate early incorporation into biomarker-informed systemic therapy and clinical trials when available. Importantly, TMB in our cohort was measured in newly diagnosed, treatment-naive tumors before radiochemotherapy, and therefore reflects intrinsic genomic instability rather than TMZ-induced hypermutation at recurrence. Unlike acquired hypermutation linked to mismatch repair deficiency and therapeutic resistance, higher baseline TMB in primary TGs independently predicted poorer outcome, suggesting that it may mark inherently aggressive biology, potentially associated with greater clonal complexity or replication stress.

Key strengths of this study include its scale for a rare anatomic location, inclusion of both pediatric and adult patients, and the predominance of resection-derived tissue, enabling integrated perioperative phenotyping with targeted panel sequencing and immunohistochemical validation. Analytically, we employed contemporary methods, including stability selection, penalized regression, causal-inference approaches, and calibration-oriented internal validation, to limit overfitting and enhance clinical interpretability. Important limitations include the retrospective design and susceptibility to selection and unmeasured confounding, particularly with respect to corridor selection, EOR, and mTV use. The single-center nature of a high-volume practice may constrain generalizability. We relied primarily on targeted panel sequencing without systematic methylation profiling or transcriptomic assays. Additional limitations include the limited number of *CDKN2A/B*-deleted tumors and incomplete ascertainment of longer-term recovery and patient-centered outcomes, including cognition and quality of life. All 65 cases classified as DMG were exclusively the *H3F3A* (H3.3) K27M mutation DMG; these findings may not necessarily apply to DMG with EZHIP overexpression and certain H3-wild-type midline gliomas with EGFR or ACVR1 alterations.

In summary, this large integrated TGs series defines the clinical, molecular, and perioperative risk landscape of a highly challenging tumor group. Achieving GTR/STR, considering synchronous mTV in high-risk anatomy, and selecting corridors tailored to baseline motor status and tract anatomy can meaningfully affect both functional outcomes and survival. The mutual exclusivity between *H3F3A* mutation and *CDKN2A/B* co-deletion and the distinct profiles of DMG versus non-DMG tumors also suggest differential therapeutic vulnerabilities (eg, epigenetic strategies versus *CDK4/6* inhibition in selected subsets). These data provide a practical framework for risk stratification and operative planning and a platform for future multicenter studies integrating high-quality surgery with rational systemic therapy in this historically intractable disease.

## Conclusion

In this cohort of 106 surgically treated TGs, we defined the clinical, radiological, and molecular landscape and showed that *H3F3A* mutation and *CDKN2A/B* co-deletion are largely mutually exclusive. More extensive resection (GTR/STR vs PR) was associated with longer OS, indicating that deep thalamic location alone should not preclude maximal safe resection. Perioperative CSF management proved equally critical: preoperative and postoperative hydrocephalus were common, and intraoperative mTV significantly reduced postoperative hydrocephalus and the need for shunting. Together, these data support an integrated strategy combining maximal safe resection, proactive CSF pathway management, and molecularly guided adjuvant therapy for patients with TG.

## Supplementary Material

noag146_Supplementary_Data

## Data Availability

The raw sequencing data generated in this study are not publicly available due to regulations concerning the protection of patient privacy. However, the data are available from the corresponding author upon reasonable request and institutional approval. All other data supporting the findings of this study are available within the article and its supplementary materials.
