# Supplementary material for: The clinical and molecular landscape of thalamic glioma
Source: Neuro Oncol. 2026 Jun 30;28(10):2417–34. doi: 10.1093/neuonc/noag146 (PMC13635587; doi:10.1093/neuonc/noag146)
Supplement: Supplementary file 1 [file noag146_supplementary_data.zip › Supplementary_Text_Clean.docx]

# 1 Methods

## 1.1 Assessment of motor function and postoperative hemiparesis

Limb motor strength was evaluated pre- and postoperatively using the Medical Research Council (MRC) 0–5 scale for each side. For analysis, the “worst” MRC grade was defined as the minimum of bilateral limb scores, and the change in strength (ΔMRC) as postoperative minus preoperative worst grade. Pre- and postoperative hemiparesis were coded as binary variables based on neurological examination.

## 1.2 Imaging and CSF flow assessment

Pre- and postoperative CT and MRI scans were retrieved from the institutional PACS and anonymized before review. Standard axial, sagittal and coronal T1-weighted post-contrast and T2-weighted sequences were used to assess tumor location, ventricular size and signs of hydrocephalus. In a subset of patients, cardiac-gated phase-contrast cine MRI of the cerebral aqueduct was acquired according to vendor-specific protocols. Time–velocity curves of cerebrospinal fluid (CSF) flow in the mid-aqueduct were generated, with positive velocities defined as flow from the third to the fourth ventricle and negative velocities as reverse flow.

## 1.3 Targeted sequencing and primary data processing

Tumor DNA was analyzed using a hybrid-capture targeted sequencing panel covering recurrently altered genes in gliomas. Over the study period, two closely related versions of this panel were used, which shared the core set of glioma-associated genes. For cases with available raw data, paired-end FASTQ files (gzip-compressed) were generated for each sample. Raw reads underwent quality assessment with FastQC, and cohort-level summaries were compiled with MultiQC. Adapter trimming and removal of low-quality bases were performed using fastp with automatic adapter detection for paired-end libraries. The resulting clean reads were used for all downstream analyses.

For a small subset of patients, only panel-based variant summary files with genomic coordinates and allele-frequency information were available; these were harmonised with the in-house call set as described below.

## 1.4 Read alignment, duplicate removal and coverage metrics

Clean reads were aligned to the human reference genome hg19/GRCh37 using bwa-mem2 with sample-specific read group tags. Alignments were sorted and indexed with samtools, and basic mapping statistics were obtained with samtools flagstat. PCR and optical duplicates were marked and removed using Picard MarkDuplicates. Target capture performance and depth of coverage across the panel were evaluated with Picard CollectHsMetrics using the panel-specific interval list.

## 1.5 Somatic SNV/indel calling and functional annotation

Somatic single-nucleotide variants (SNVs) and small insertions/deletions (indels) were called on duplicate-filtered BAM files using the GATK Mutect2 pipeline in tumor-only mode. Calling was restricted to the panel target regions and incorporated a germline reference resource from gnomAD as well as a panel of normals to reduce sequencing artifacts. Orientation bias models were learned using GATK LearnReadOrientationModel, and candidate variants were filtered with GATK FilterMutectCalls followed by GATK FilterAlignmentArtifacts.

High-confidence calls were further retained by applying hard filters that required a total read depth ≥ 100, a variant allele fraction (VAF) ≥ 0.03 and a tumor log-odds score ≥ 6. Resulting variants were annotated with the Ensembl Variant Effect Predictor (VEP) on GRCh37, adding gene symbol, canonical transcript, functional consequence terms, protein-level HGVS notations, and population allele frequencies.

## 1.6 Variant curation, integration and definition of non-synonymous events

Annotated somatic variants derived from the above pipeline were curated and combined into a unified call set. For each variant, allele depths were parsed to obtain reference and alternate read counts, and VAF was calculated from the reported allele fraction or from the ratio of alternate reads to total depth. Variants were retained for downstream analyses if they met all of the following criteria: total depth ≥ 30, at least 5 supporting alternate reads and VAF ≥ 0.05.

For the small subset of cases with only panel-level variant summaries available, we extracted genomic coordinates, reference and alternate alleles, gene symbols, functional consequence labels and allele-frequency information from the reports, converted allele frequencies to VAF, and applied the same depth and VAF thresholds as above.

Functional consequence terms from VEP were mapped to standard Mutation Annotation Format (MAF) classes (e.g. missense, nonsense, frameshift, in-frame, splice-site, untranslated region, intronic, intergenic). Intergenic variants were removed except for *TERT* promoter mutations, which were retained as IGR events. Gene symbols were harmonized, and a panel of hypermutable or low-specificity genes (such as *MUC16*, *TTN* and other known background genes) was excluded. Duplicated entries were collapsed by genomic position and alleles. The resulting curated dataset was stored in a MAF-like structure and used for all mutation-level analyses.

## 1.7 Tumor mutational burden (TMB)

Panel-based tumor mutational burden was estimated by counting nonsynonymous coding variants per megabase of captured sequence. The effective size of the targeted region was calculated from the panel design by merging overlapping intervals and summing their lengths. For each sample, we counted protein-altering exonic variants (including missense, frameshift, nonsense, start-lost, canonical splice-site and in-frame indels) that passed stringent read-level filters (depth ≥ 100 and VAF ≥ 0.05). TMB was defined as the number of such mutations divided by the panel size and expressed as mutations per megabase (mut/Mb).

## 1.8 Copy-number variation (CNV) analysis

Copy-number alterations were inferred from read-depth data using the GATK CNV workflow. Interval-level read counts across the panel targets were obtained with GATK CollectReadCounts, restricting to reads with mapping quality ≥ 20. Counts were denoised using GATK DenoiseReadCounts, with a panel of normals applied when available, and segmented using GATK ModelSegments. Copy-ratio segments were then classified with GATK CallCopyRatioSegments, yielding per-sample segmentation files with log2 copy ratios.

To derive gene-level copy-number calls, these segments were intersected with a curated list of panel genes using bedtools. For each gene in each sample, a length-weighted mean log2 copy ratio was calculated and converted to an absolute copy-number estimate assuming a diploid baseline. Genes with estimated copy number > 3 were considered amplifications (AMP), and those with copy number < 1 were considered deletions (DEL). The resulting long-format gene-level CNV table was used for CNV-focused analyses and visualization.

## 1.9 Clinical data and integrative mutation–CNV visualisation

Clinical and pathological variables (age group, diagnosis category, sex, WHO grade, 1p/19q co-deletion status and MGMT promoter methylation status) were retrieved from a curated clinical database and matched to sequencing samples via patient identifiers. Age-based subgroups were defined as pediatric and adult; diagnostic subgroups were defined as diffuse midline glioma (DMG) and non-DMG (non-DMG).

Curated mutation and CNV tables were imported into R and converted into MAF-like objects using maftools. Non-synonymous variant classes (frame-shift, in-frame indel, missense, nonsense, splice-site, translation start-site and selected IGR events) were used for mutation-level visualisation. Integrated waterfall plots (oncoplots) were generated to display, for each sample, both SNV/indel events and gene-level CNVs in predefined glioma-relevant genes, with multiple clinical variables shown as annotation bars. Separate oncoplots were produced for pediatric versus adult tumors and for DMG versus non-DMG to compare subgroup-specific genomic patterns.

## 1.10 Pairwise co-alteration analysis

Targeted sequencing data were converted into a binary alteration matrix, coding recurrent SNVs, amplifications and deletions of *H3F3A*, *CDKN2A*/B and other panel genes as present or absent in each tumor. For every gene pair, we calculated the observed number of double-positive cases and the expected number under an independence assumption, and summarized the deviation as the log₂ ratio of observed versus expected counts. Positive values were interpreted as enrichment (co-occurrence) and negative values as depletion (mutual exclusivity). Two-sided Fisher’s exact tests were applied to 2×2 contingency tables for each gene pair, and P values were adjusted for multiple comparisons using the Benjamini–Hochberg procedure.

## 1.11 Molecular axis and RTK/PI3K pathway categories

Based on key driver events, tumors were classified into three mutually exclusive molecular “axes”: an H3K27 axis was defined by the presence of an *H3F3A* K27M mutation; a 9p21/cell-cycle axis was defined by deletion of *CDKN2A* and/or *CDKN2B* in the absence of *H3F3A* mutation; all remaining cases were assigned to a Neither group. In parallel, RTK/PI3K pathway status was coded as altered if any alteration was present in *EGFR, NF1, PIK3CA, PIK3R1, PTEN, AKT1, RICTOR* or *FGFR1*, and as intact otherwise. The joint distribution of diagnosis category (DMG vs non-DMG), molecular axis and RTK/PI3K status was summarized as case counts and visualized using alluvial–type plots.

## 1.12 Diagnostic performance of 9p21 loss for non-DMG

Analyses focusing on H3K27M and 9p21 status were restricted to cases with complete information on *H3F3A* and *CDKN2A/B.* 9p21 loss was defined as deletion of *CDKN2A* and/or *CDKN2B*. Treating diagnosis (non-DMG vs DMG) as the reference standard and 9p21 loss (present vs absent) as the index test, we constructed a 2×2 contingency table and calculated sensitivity, specificity, positive and negative predictive values (PPV and NPV), as well as positive and negative likelihood ratios (LR⁺ and LR⁻). Exact 95% confidence intervals for proportions were derived from binomial models, and confidence intervals for likelihood ratios were obtained from log-transformed estimates.

## 1.13 Single-cell RNA sequencing and preprocessing

Fresh tumor samples from one unilateral thalamic DMG and one unilateral thalamic non-DMG underwent droplet-based single-cell RNA sequencing. Raw gene–cell count matrices were corrected for ambient RNA and cell barcodes were filtered using a dedicated background-removal tool (CellBender). Downstream analyses were performed in R (Seurat V5 framework). Low-quality cells were excluded if they had <500 or >6,000 detected genes, <1,000 UMIs, >20% mitochondrial transcripts, or >3% hemoglobin transcripts. Genes on a curated blacklist (e.g., ribosomal, mitochondrial pseudogenes and others prone to ambient contamination) were removed. Cell-cycle scores were computed using canonical S-phase and G2/M gene sets and stored as metadata.

To remove doublets, we performed an initial PCA and graph-based clustering for each sample and then applied DoubletFinder with data-driven choice of pK and expected doublet rate. Cells classified as doublets were discarded, and only high-quality singlets were carried forward.

## 1.14 Normalization, integration and clustering

Filtered count matrices from the two tumors were merged into a single Seurat object and normalized using SCTransform (glmGamPoi), regressing out mitochondrial and hemoglobin percentages as well as S and G2/M cell-cycle scores. Principal component analysis (PCA) was performed on the normalized data, and the two tumors were integrated using Seurat’s reciprocal PCA (RPCA) integration pipeline with SCT normalization. On the integrated embedding, we recalculated PCA, constructed a shared nearest-neighbor graph using the leading PCs, and performed graph-based clustering with a resolution of 1.6. UMAP and t-SNE were computed on the integrated PCA space for visualization.

## 1.15 Inference of copy-number alterations and malignant cell calling

Copy-number alterations (CNAs) were inferred from single-cell transcriptomes using two complementary approaches. First, copyKAT was run on raw UMI matrices from each sample to classify cells as “aneuploid” or “diploid/normal” and to derive a continuous copyKAT score. Second, inferCNV was applied using non-malignant reference populations to obtain per-cell CNV intensity profiles and a quantitative CNV score. For downstream visualization, copyKAT and inferCNV scores were averaged at the cluster level and used to stratify clusters into CNV-high (malignant) versus CNV-low (non-malignant) compartments. To generate genome-wide CNV panoramas, inferCNV log2 copy-number intensities from malignant cells were binned along the genome and averaged per chromosome.

## 1.16 Cell-type annotation and composition analysis

Clusters were annotated based on canonical marker genes and differential expression. Marker discovery combined COSG and Wilcoxon rank-sum testing (FindAllMarkers). Clusters expressing *PTPRZ1, PTN* and *SOX5* were annotated as malignant glioma cells; *TYROBP*, *AIF1* and *C1QA* as myeloid cells; *CD3D, TRBC2* and *GZMA* as T/NK cells; *CLDN5, VWF* and *ESAM* as endothelial cells; and *MOBP, MOG* and *CLDN11* as normal oligodendrocytes. The proportion of each major cell type was calculated for the DMG and non-DMG tumors, and stacked bar plots were used to compare tumor composition between the two diagnoses.

## 1.17 Raw-window and core-window Δlog2 metrics

Counts were library-size normalised and log-transformed to obtain gene-level expression values in malignant and reference cells. For chr9:21–23 Mb, an automatically expanded “raw window” (starting from the pre-defined core and extended until ≥15 genes) was used to calculate, per sample, the fraction of malignant cells with a mean Δlog2 (observation vs reference) below −0.20. In parallel, core-window genes within chr9:21–23 Mb were summarised as per-gene mean Δlog2 and the proportion of malignant cells below the same threshold.

## 1.18 Run-length and core-4 anchoring metrics

Using Δlog2 values re-centred by the median of chr9 flanking genes, we quantified the fraction of malignant cells carrying at least one run of ≥3 or ≥4 consecutive core-window genes below the chosen cut-offs. A complementary “core-4 anchored” metric recorded, for each sample, the proportion of malignant cells in which at least 4 of 5 core genes (*KLHL9, MTAP, CDKN2A, CDKN2B-AS1, CDKN2B*) showed Δlog2 below −0.25.

## 1.19 Variable selection for postoperative hydrocephalus

To screen potential predictors of postoperative hydrocephalus, we applied stability selection with LASSO logistic regression to the full set of preoperative clinical, radiological and molecular variables. In each of 1000 iterations, a random 80% subsample of patients was drawn without replacement and a binomial LASSO model was fitted with K-fold cross-validation (typically 10 folds) to select the penalty parameter. Variables with non-zero coefficients at the cross-validated penalty levels were recorded and their selection frequencies were summarised across subsamples.

## 1.20 Firth penalised logistic regression

Variables selected a priori and informed by the stability-selection step were entered into a multivariable Firth penalised logistic regression model for postoperative hydrocephalus (Yes vs No). Regression coefficients were estimated on the log-odds scale, with odds ratios (ORs) and 95% confidence intervals (CIs) obtained by exponentiation. Wald-type tests were used for hypothesis testing, and model estimates were displayed in a forest-plot format.

## 1.21 Model discrimination and calibration

Discriminative performance of the Firth model was quantified using the area under the receiver operating characteristic curve (AUC) with 95% CIs from DeLong’s method. Internal validation was performed by bootstrap optimism correction and by estimating out-of-bag AUC from repeated resampling. Model calibration was evaluated using logistic recalibration (calibration intercept and slope), decile-based calibration plots and a smoothed LOESS calibration curve.

## 1.22 Propensity score estimation and ATT analysis for mTV

To estimate the effect of mTV on postoperative hydrocephalus, we used propensity score (PS)–based methods with mTV as the exposure. PSs for receiving mTV were estimated from prespecified baseline covariates (including demographic, clinical, radiological and molecular factors, as well as preoperative hydrocephalus, performance status and surgical approach) using ridge-penalised logistic regression as the main specification, and standard logistic regression and covariate-balancing propensity scores (CBPS) as sensitivity specifications. For the average treatment effect in the treated (ATT), we implemented full matching on the PS, with exact matching on key variables (such as preoperative hydrocephalus and surgical approach) when feasible. Covariate balance after matching was assessed using standardised mean differences and effective sample size.

## 1.23 Overlap weighting and ATO analysis for mTV

For the average treatment effect in the overlap population (ATO), we constructed overlap weights from the PS, assigning weights proportional to 1–PS for treated patients and PS for controls, with optional stabilisation. Separate overlap-weighted analyses were conducted for each PS specification (ridge-based, conventional logistic and CBPS). Under each weighting scheme, we estimated weighted risks, risk differences, risk ratios and odds ratios for postoperative hydrocephalus. Robust variance estimation and effective-sample-size–based corrections were used where appropriate, and E-values were calculated for selected risk-ratio estimates as a measure of sensitivity to unmeasured confounding.

## 1.24 Difference-in-differences framework

To incorporate paired pre- and postoperative hydrocephalus status, we specified a two-period difference-in-differences (DID) design, treating time (preoperative vs postoperative) as the period and mTV as the treatment. Binary hydrocephalus status was coded at each timepoint and the data were reshaped into long format with one record per patient-period. The main DID model included patient fixed effects, a post-period indicator and its interaction with mTV, with cluster-robust standard errors based on patient-level clustering. The interaction term was interpreted as the DID contrast in the probability of hydrocephalus between mTV and non-mTV patients.

## 1.25 Sensitivity analyses for the DID estimand

Robustness of the DID estimand was examined using several complementary approaches. First, wild cluster bootstrap procedures with Rademacher weights were applied to obtain bootstrap-based CIs for the interaction term. Second, randomisation-inference procedures were implemented by reassigning pseudo-mTV labels within anatomically comparable patient pools. Third, placebo DID analyses were performed within the non-mTV group by assigning pseudo-treatment indicators, to assess potential violations of the DID assumptions in the absence of mTV.

## 1.26 Overall survival and Kaplan–Meier analyses

Overall survival (OS) was defined as the interval from the date of histopathological diagnosis to death from any cause; patients alive or lost to follow-up were censored at the date of last contact. Kaplan-Meier curves were generated for predefined clinical, treatment-related and molecular subgroups, including diffuse midline glioma status, age group (adult vs pediatric), tumor mutational burden (high vs low, dichotomized at the cohort median), MGMT promoter status, extent of resection (GTR/STR vs PR), surgical approach, presence of pre- and postoperative hydrocephalus or hemiparesis, pre- and postoperative Karnofsky Performance Status (KPS; <60 vs ≥60), *TP53* and *TERT* mutation status, and *CDKN2A* and *CDKN2B* deletion status. Group differences in OS were compared using two-sided log-rank tests.

## 1.27 Cox proportional hazards modeling

For prognostic modeling of OS, univariable Cox proportional hazards models were first fitted for each candidate variable. A multivariable Cox model was then specified a priori including extent of resection (PR vs GTR/STR), TMB group (high vs low), postoperative KPS group (<60 vs ≥60), and diffuse midline glioma status (DMG vs non-DMG). Hazard ratios (HRs) and 95% confidence intervals were estimated, and the proportional hazards assumption was assessed using Schoenfeld residuals. All time-to-event analyses were performed in R (survival and survminer packages).

**Supplementary Figures&Tables Legends**

**Figure S1. Integrated mutational and copy-number landscape of thalamic gliomas (TGs) stratified by age.**

Oncoplot of somatic SNVs/indels (top) and copy-number alterations (CNVs; bottom) across 106 TGs, stratified by age group (adult, n = 87; pediatric, n = 19). Columns represent individual tumors and rows represent selected recurrently altered genes. Mutation classes and CNV states (amplification vs deletion) are color-coded. Annotation tracks summarize MGMT promoter methylation status, 1p/19q codeletion, WHO grade, sex, diagnosis, and age group. Percentages shown beside each gene indicate the corresponding alteration frequencies for SNVs/indels or CNVs. Genes highlighted in red denote significantly different alteration frequencies between adult and pediatric tumors (Fisher’s exact test, *P* < 0.05).

**Figure S2. Single-cell transcriptomic landscape, CNV-based classification, and 9p21.3 expression-based loss metrics in DMG and non-DMG thalamic gliomas.**

(A) UMAP embedding of all cells colored by diagnosis (DMG vs non-DMG).

(B) Same UMAP colored by copyKAT classification (aneuploid, diploid/normal or NA).

(C) Same UMAP colored by inferCNV-derived CNV load (CNV-high vs CNV-low).

(D) Dot plot showing, for each cluster, the scaled average expression (color) and percentage of expressing cells (dot size) of canonical marker genes for malignant cells (*PTPRZ1*, *PTN*, *SOX5*), myeloid cells (*TYROBP*, *AIF1*, *C1QA*), T/NK cells (*CD3D*, *TRBC2*, *GZMA*), endothelial cells (*CLDN5*, *VWF*, *ESAM*), and normal oligodendrocytes (*MOBP*, *MOG*, *CLDN11*). Right-hand columns depict cluster-level mean inferCNV and copyKAT scores.

(E) Genome-wide CNV panoramas (log2 CNV) for malignant cells from the DMG and non-DMG tumors, binned along the genome by chromosome.

(F,G) UMAP (F) and t-SNE (G) embeddings colored by cell type (malignant cells, myeloid cells, T/NK cells, endothelial cells and normal oligodendrocytes).

(H) Stacked bar plot showing the relative proportion of each major cell type in the DMG and non-DMG samples.

(I-K) Detailed single-cell expression-based metrics of 9p21.3 loss in malignant cells from the DMG and non-DMG tumors: (I) fraction of cells below the Δlog2 threshold within the chr9:21–23 Mb window; (J) core-window per-gene Δlog2 shifts highlighting *KLHL9*, *MTAP*, *CDKN2A*, *CDKN2B-AS1* and *CDKN2B*; (K) run-length support metrics.

**Figure S3. Clinical examples illustrating the impact of postoperative hydrocephalus with and without mTV.**

(A) Preoperative CT and contrast-enhanced MR images of a patient with a right thalamic glioma showing ventricular enlargement and periventricular edema consistent with obstructive hydrocephalus.

(B) Early postoperative CT and MRI from the same patient, demonstrating near-total tumor resection with partial resolution of periventricular changes but persistent ventricular dilatation.

(C) CT and follow-up contrast-enhanced MRI obtained after ventriculo-peritoneal shunt placement, showing the intraventricular catheter and valve artefacts, decreased ventricular size and stable postoperative tumor bed.

(D) Preoperative phase-contrast cine MRI time-velocity curve of cerebrospinal fluid flow in the mid-aqueduct over the cardiac cycle in a second patient with a right thalamic glioma, demonstrating an irregular disturbed waveform.

(E) Postoperative aqueductal time-velocity curve in the same patient after tumor resection and mTV, showing persistent but altered abnormalities in CSF flow dynamics.

(F) Corresponding preoperative CT and MRI in this second patient, illustrating supratentorial hydrocephalus and focal compression of the aqueduct by the thalamic tumor.

(G) Intraoperative and postoperative images after mTV and tumor removal in the same patient, demonstrating the ventriculostomy, resolution of periventricular edema, and restoration of the aqueductal lumen without the need for a ventriculo-peritoneal shunt.

**Figure S4. Kaplan-Meier analyses of overall survival according to selected molecular alterations.**

(A) *TP53* mutation status (mutant vs wildtype).

(B) *TERT* mutation status (mutant vs wildtype).

(C) *CDKN2A* deletion status (deletion vs wildtype).

(D) *CDKN2B* deletion status (deletion vs wildtype).

For each panel, Kaplan-Meier curves display overall survival in months with corresponding log-rank P values and numbers at risk shown below the x-axis.

**Table S1. The detailed summary of all samples in which BRAF-related variants were detected.**

**Table S2. ATT of mTV on postoperative hydrocephalus after propensity-score full matching.**

Abbreviations: mTV, microsurgical third ventriculostomy; RD, risk difference; RD_unadj, unadjusted RD; RD_ms, RD from marginal standardization (primary estimand); bootCI, 95% bootstrap confidence interval; OR, odds ratio; RR, risk ratio. Firth OR was obtained from weighted Firth penalized logistic regression; RR (Haldane) uses a 0.5 continuity correction. The E-value (protective) is the minimum strength of association (on the risk-ratio scale) that an unmeasured confounder would need with both mTV and postoperative hydrocephalus to fully explain away the observed effect. Negative RD and values <1 for OR and RR indicate lower risk in the mTV group.

**Table S3. Sensitivity analyses of the ATT of mTV on postoperative hydrocephalus.**

Methods: 1, full matching with ridge propensity scores (λ = λ_min), exact matching as in the main analysis; 2, nearest-neighbor 1:4 with replacement on ridge PS (λ = λ_min); 3, full matching on logistic-regression PS; 4, full matching on CBPS-derived PS; 5, full matching on ridge PS (λ = λ_1se), discarding both tails; 6, nearest-neighbor 1:2 with replacement on logistic PS, caliper = 0.20·SD(logit PS), discarding treated units outside the caliper; 7, nearest-neighbor 1:4 with replacement on ridge PS (λ = λ_min), caliper = 0.05 on the probability scale, discarding treated units outside the caliper; 8, nearest-neighbor Mahalanobis 1:2 with replacement, exact matching as in the main analysis; 9, nearest-neighbor Mahalanobis 1:4 with replacement, exact matching as in the main analysis.

**Table S4. ATO of mTV on postoperative hydrocephalus after overlap weighting.**

Abbreviations: ATO, average treatment effect in the overlap population; PS, propensity score; RD_ms, risk difference from marginal standardization (primary estimand); OR, odds ratio; RR, risk ratio. Firth OR was obtained from weighted Firth penalized logistic regression; RR (Haldane) uses a 0.5 continuity correction. Negative RD and values <1 for OR and RR indicate a lower risk of postoperative hydrocephalus in the mTV group.

**Table S5. DID estimates of the effect of mTV on postoperative hydrocephalus.**

Abbreviations: DID, difference-in-differences; FE, fixed effects; SE, standard error. Negative Δ indicates an additional reduction in postoperative hydrocephalus associated with mTV (protective effect).

**Table S6. Cox regression analysis for overall survival.**

Reference categories: GTR/STR, low TMB, postoperative KPS ≥ 60, non-DMG.

**Table S7. Summary of stratified survival analyses in the DMG and non-DMG cohorts.**
