# Supplementary material for: The clinical and molecular landscape of thalamic glioma
Source: Neuro Oncol. 2026 Jun 30;28(10):2417–34. doi: 10.1093/neuonc/noag146 (PMC13635587; doi:10.1093/neuonc/noag146)
Supplement: Supplementary file 1 [file noag146_supplementary_data.zip › Supplementary_Tables_Clean.docx]

**Table S1. The detailed summary of all samples in which BRAF-related variants were detected.**

| **Patient ID** | **DMG status** | **WHO grade** | **BRAF category** | **Protein change** | **Genomic context** |
| --- | --- | --- | --- | --- | --- |
| 24220 | DMG | 4 | V600E | p.V600E | Missense_Mutation |
| 23691 | DMG | 4 | V600E | p.V600E | Missense_Mutation |
| 17085 | DMG | 4 | V600E | p.V600E | Missense_Mutation |
| 21180 | DMG | 4 | non-hotspot missense | p.D594G | Missense_Mutation |
| 22909 | non-DMG | 4 | non-hotspot missense | p.D7N | Missense_Mutation |
| 22023 | non-DMG | 4 | non-hotspot missense | p.V28E | Missense_Mutation |
| 19683 | non-DMG | 4 | intronic variant only | - | Intron |
| 15532 | DMG | 4 | intronic variant only | - | Intron |
| 12855 | non-DMG | 4 | intronic variant only | - | Intron |
| 12647 | DMG | 4 | intronic variant only | - | Intron |
| 12244 | non-DMG | 4 | intronic variant only | - | Intron |
| 12561 | non-DMG | 4 | intronic variant only | - | Intron |
| 19553 | DMG | 4 | intronic variant only | - | Intron |
| 19249 | non-DMG | 4 | intronic variant only | - | Intron |
| 17876 | DMG | 4 | intronic variant only | - | Intron |
| 14922 | DMG | 4 | intronic variant only | - | Intron |
| 23795 | DMG | 4 | intronic variant only | - | Intron |
| 17299 | non-DMG | 4 | intronic variant only | - | Intron |
| 17700 | non-DMG | 4 | intronic variant only | - | Intron |
| 18676 | DMG | 4 | intronic variant only | - | Intron |
| 29343 | DMG | 4 | intronic variant only | - | Intron |
| 32999 | DMG | 4 | intronic variant only | - | Intron |
| 35146 | non-DMG | 4 | intronic variant only | - | Intron |
| 29723 | DMG | 4 | intronic variant only | - | Intron |
| 29733 | non-DMG | 4 | intronic variant only | - | Intron |
| 29748 | non-DMG | 2 | intronic variant only | - | Intron |
| 30066 | DMG | 4 | intronic variant only | - | Intron |
| 30083 | DMG | 4 | intronic variant only | - | Intron |
| 30793 | non-DMG | 4 | intronic variant only | - | Intron |
| 31558 | DMG | 4 | intronic variant only | - | Intron |
| 32124 | DMG | 4 | intronic variant only | - | Intron |
| 33628 | non-DMG | 4 | intronic variant only | - | Intron |
| 34437 | non-DMG | 4 | intronic variant only | - | Intron |
| 34706 | DMG | 4 | intronic variant only | - | Intron |
| 34826 | non-DMG | 4 | intronic variant only | - | Intron |
| 35050 | non-DMG | 4 | intronic variant only | - | Intron |

**Table S2. ATT of mTV on postoperative hydrocephalus after propensity-score full matching.**

| **Estimand** | **Risks** | **RD_unadj** | **RD_ms**  **(primary)** | **Firth OR** | **RR**  **(Haldane)** | **E-value**  **(protective)** |
| --- | --- | --- | --- | --- | --- | --- |
| ATT | mTV/Control (%) | value (95%CI; *P*) | Value (95%CI; bootCI) | Value (95%CI; *P*) | value | value |
|  | 0.0/58.2 | -0.582 (-1.011, -0.153; 0.016) | -0.609 (-0.700, -0.517; -0.870, -0.000) | 0.01 (0.00, 0.24; 0.0016) | 0.096 | 20.34 |

Abbreviations: mTV, microsurgical third ventriculostomy; RD, risk difference; RD_unadj, unadjusted RD; RD_ms, RD from marginal standardization (primary estimand); bootCI, 95% bootstrap confidence interval; OR, odds ratio; RR, risk ratio. Firth OR was obtained from weighted Firth penalized logistic regression; RR (Haldane) uses a 0.5 continuity correction. The E-value (protective) is the minimum strength of association (on the risk-ratio scale) that an unmeasured confounder would need with both mTV and postoperative hydrocephalus to fully explain away the observed effect. Negative RD and values <1 for OR and RR indicate lower risk in the mTV group.

**Table S3. Sensitivity analyses of the ATT of mTV on postoperative hydrocephalus.**

| **Method** | **Risk_mTV_%** | **Risk_Control_%** | **RD_ms** | **RD_ms_CI** | **Boot_RD_CI** | **Firth_OR** | **Firth_OR_CI** | **Firth_OR_*P*** | **Haldane_RR** | **Evalue_protective** |
| --- | --- | --- | --- | --- | --- | --- | --- | --- | --- | --- |
| 1 | 0 | 61.7 | -0.628 | (-0.759, -0.498) | (-0.821, -0.133) | 0.06 | (0.00, 0.51) | 0.007548 | 0.091 | 21.56 |
| 2 | 0 | 68.8 | -0.619 | (-0.704, -0.535) | (-1.000, -0.000) | 0.05 | (0.00, 0.50) | 0.007564 | 0.082 | 23.79 |
| 3 | 0 | 32.8 | -0.381 | (-0.549, -0.214) | (-0.853, -0.076) | 0.04 | (0.00, 0.97) | 0.04664 | 0.167 | 11.47 |
| 4 | 0 | 49.7 | -0.518 | (-0.645, -0.391) | (-0.825, -0.110) | 0.07 | (0.00, 0.86) | 0.03442 | 0.112 | 17.39 |
| 5 | 0 | 58.2 | -0.609 | (-0.700, -0.517) | (-0.863, -0.001) | 0.01 | (0.00, 0.24) | 0.001584 | 0.096 | 20.34 |
| 6 | 0 | 50 | -0.5 | (-0.500, -0.500) | (-0.917, 0.000) | 0.15 | (0.00, 1.73) | 0.1369 | 0.2 | 9.47 |
| 7 | 0 | 75 | -0.8 | (-0.800, -0.800) | (-1.000, 0.000) | 0.11 | (0.00, 6.77) | 0.3063 | 0.357 | 5.04 |
| 8 | 0 | 68.8 | -0.634 | (-0.734, -0.533) | (-0.933, 0.021) | 0.08 | (0.00, 1.13) | 0.06126 | 0.083 | 23.68 |
| 9 | 0 | 68.8 | -0.696 | (-0.911, -0.482) | (-0.902, -0.210) | 0.08 | (0.00, 0.70) | 0.02008 | 0.082 | 23.84 |

Methods: 1, full matching with ridge propensity scores (λ = λ_min), exact matching as in the main analysis; 2, nearest-neighbor 1:4 with replacement on ridge PS (λ = λ_min); 3, full matching on logistic-regression PS; 4, full matching on CBPS-derived PS; 5, full matching on ridge PS (λ = λ_1se), discarding both tails; 6, nearest-neighbor 1:2 with replacement on logistic PS, caliper = 0.20·SD(logit PS), discarding treated units outside the caliper; 7, nearest-neighbor 1:4 with replacement on ridge PS (λ = λ_min), caliper = 0.05 on the probability scale, discarding treated units outside the caliper; 8, nearest-neighbor Mahalanobis 1:2 with replacement, exact matching as in the main analysis; 9, nearest-neighbor Mahalanobis 1:4 with replacement, exact matching as in the main analysis.

**Table S4. ATO of mTV on postoperative hydrocephalus after overlap weighting.**

| **PS / weighting scheme** | **Risk in mTV / control (%)** | **RD_ms**  **(primary, 95% CI)** | **Firth OR**  **(95% CI; *P*)** | **RR**  **(Haldane)** | **E-value**  **(protective)** |
| --- | --- | --- | --- | --- | --- |
| CBPS PS, overlap weighting | 0.0 / 62.4 | -0.667 (-0.834, -0.501) | 0.00 (0.00, 0.01; *P* ≈ 0.00)* | 0.014 | 137.79 |
| Ridge PS (λ = min), overlap weighting | 0.0 / 49.3 | -0.478 (-0.597, -0.358) | 0.01 (0.00, 0.11; *P* = 1.06×10⁻⁸) | 0.019 | 106.09 |
| GLM PS, overlap weighting | 0.0 / 61.2 | -0.626 (-0.775, -0.476) | 0.00 (0.00, 0.02; *P* = 4.89×10⁻¹5) | 0.015 | 131.30 |

Abbreviations: ATO, average treatment effect in the overlap population; PS, propensity score; RD_ms, risk difference from marginal standardization (primary estimand); OR, odds ratio; RR, risk ratio. Firth OR was obtained from weighted Firth penalized logistic regression; RR (Haldane) uses a 0.5 continuity correction. Negative RD and values <1 for OR and RR indicate a lower risk of postoperative hydrocephalus in the mTV group.

**Table S5. DID estimates of the effect of mTV on postoperative hydrocephalus.**

| **Analysis / procedure** | **Δ (DID estimate)** | **95% CI** | ***P*-value (or summary)** | **Comment** |
| --- | --- | --- | --- | --- |
| Patient FE, cluster-robust SEs (main DID) | -0.735 | -0.878 to -0.591 | 5.44 × 10⁻¹⁷ | Main DID analysis |
| Wild cluster bootstrap (Rademacher, B = 9,999) | -0.735 | -0.841 to -0.629 | 0.0008 | Small-sample robust CI and p-value |
| Same-approach subset | -0.692 | -0.903 to -0.482 | 6.77 × 10⁻⁸ | Restricting to same surgical approach |
| Leave-one-out (mTV patients, n = 8) | -0.735 | — | Each exclusion: *P* ≈ 6.0 × 10⁻¹⁷ | No single mTV patient drives result |
| Randomization inference (B = 5,000) | -0.735 | — | Empirical *P* = 0.0008 | Permutation-based *P*-value |
| Placebo DID within controls | Mean Δ = -0.110 | SD 0.163; q₂.₅/₅₀/₉₇.₅: -0.392, -0.119, 0.153 | Empirical *P* ≈ 1.00 vs observed | Null distribution centred near zero |

Abbreviations: DID, difference-in-differences; FE, fixed effects; SE, standard error. Negative Δ indicates an additional reduction in postoperative hydrocephalus associated with mTV (protective effect).

**Table S6. Cox regression analysis for overall survival.**

| **Variable** | **Univariable HR**  **(95% CI)** | **Univariable *P*** | **Multivariable HR**  **(95% CI)** | **Multivariable *P*** |
| --- | --- | --- | --- | --- |
| Extent of resection (PR vs GTR/STR) | 6.31 (3.12–12.8) | **<0.001** | 5.88 (2.81–12.3) | **<0.001** |
| TMB (High vs Low) | 2.17 (1.18–3.99) | **0.013** | 2.02 (1.06–3.87) | **0.033** |
| Postoperative KPS (<60 vs ≥60) | 2.44 (1.27–4.71) | **0.008** | 1.73 (0.81–3.68) | 0.158 |
| DMG status (DMG vs non-DMG) | 1.35 (0.73–2.52) | 0.336 | 1.24 (0.65–2.39) | 0.513 |

Reference categories: GTR/STR, low TMB, postoperative KPS ≥ 60, non-DMG.

**Table S7. Summary of stratified survival analyses in the DMG and non-DMG cohorts.**

| Stratum | Variable | Contrast | Log-rank *P* value | Hazard ratio | 95% CI | Cox *P* value |
| --- | --- | --- | --- | --- | --- | --- |
| DMG | Postoperative KPS | <60 vs ≥60 | 0.12 | 2.02 | 0.82–4.98 | 0.128 |
| DMG | Extent of resection | PR vs GTR/STR | 0.0001 | 4.72 | 2.01–11.09 | 0.0004 |
| DMG | TMB | High vs Low | 0.059 | 2.13 | 0.96–4.71 | 0.062 |
| non-DMG | Postoperative KPS | <60 vs ≥60 | 0.0062 | 4.25 | 1.45–12.45 | 0.0085 |
| non-DMG | Extent of resection | PR vs GTR/STR | <0.0001 | 10.31 | 2.94–36.14 | 0.0003 |
| non-DMG | TMB | High vs Low | 0.17 | 1.97 | 0.74–5.29 | 0.176 |
